# A flexible framework for sequential estimation of model parameters in computational hemodynamics

**DOI:** 10.1186/s40323-020-00186-x

**Published:** 2020-12-02

**Authors:** Christopher J. Arthurs, Nan Xiao, Philippe Moireau, Tobias Schaeffter, C. Alberto Figueroa

**Affiliations:** 1 Dept. of Biomedical Engineering, King’s College London, London, UK; 2 Inria, Inria Saclay-Ile de France, 91128 Palaiseau, France; 3 LMS, Ecole Polytechnique, CNRS, Institut Polytechnique de Paris, 91128 Palaiseau, France; 4 Physikalisch-Technische Bundesanstalt, Berlin, Germany; 5 Technical University Berlin, Berlin, Germany; 6 Depts. of Surgery and Biomedical Engineering, University of Michigan, 2800 Plymouth Rd, Ann Arbor, MI 48109 USA

**Keywords:** Kalman filtering, Computational hemodynamics, Data assimilation, Parameter estimation, Stiffness, Boundary conditions, Patient specific modeling

## Abstract

A major challenge in constructing three dimensional patient specific hemodynamic models is the calibration of model parameters to match patient data on flow, pressure, wall motion, etc. acquired in the clinic. Current workflows are manual and time-consuming. This work presents a flexible computational framework for model parameter estimation in cardiovascular flows that relies on the following fundamental contributions. (i) A Reduced-Order Unscented Kalman Filter (ROUKF) model for data assimilation for wall material and simple lumped parameter network (LPN) boundary condition model parameters. (ii) A constrained least squares augmentation (ROUKF-CLS) for more complex LPNs. (iii) A “Netlist” implementation, supporting easy filtering of parameters in such complex LPNs. The ROUKF algorithm is demonstrated using non-invasive patient-specific data on anatomy, flow and pressure from a healthy volunteer. The ROUKF-CLS algorithm is demonstrated using synthetic data on a coronary LPN. The methods described in this paper have been implemented as part of the CRIMSON hemodynamics software package.

## Introduction

Computational models of hemodynamics are powerful tools for studying the cardiovascular system in health and disease. In particular, three-dimensional models of blood flow in the vasculature—with or without fluid-structure interaction (FSI)—have applications in non-invasive diagnostics, medical device design, surgical planning, and disease research. Due to the scarcity of direct data on flow and pressure, achieving pathophysiologically accurate results often requires specification of boundary conditions via reduced order models such as lumped parameter networks (LPN). Furthermore, in the case of FSI models, the parameters defining the structural stiffness also affect the solution greatly. It follows then that a primary challenge in constructing patient-specific models is the determination of parameters (LPN or structural stiffness) which make the simulation results agree with clinical data. Due to the high computational demand of such models, an efficient and automatic parameter estimation strategy is highly desirable.

Such parameter estimation, when based on observations of a system, is called data assimilation. Broadly, this involves combining different sources of data with a mathematical model of a physical system, in order to estimate that system’s true state. This permits the discovery of “hidden” quantities (i.e. model parameters) that may not be directly observable. In our case, the underlying mathematical model is a 3D FSI formulation of the Navier–Stokes equations, describing hemodynamics and vessel wall mechanics in an vascular geometry, and including coupled LPN boundary condition models of the downstream vasculature.

Previous studies have described algorithms for automatically identifying outflow boundary conditions and arterial wall material properties. Estimation approaches not involving the Kalman filter include the following. Grinberg and Karniadakis [1] considered a time-dependent RC-type boundary condition for imposing measured flow rates in 3D arterial tree models. Troianowski et al. [2] described an iterative fixed-point procedure for finding total resistances combined with a morphometric approach for tuning three-element Windkessel boundary conditions in 3D pulmonary arterial trees. Spilker and Taylor [3] proposed a procedure based on a quasi-Newton method to determine boundary condition parameters in a 3D model of abdominal aortic hemodynamics to match patient recordings of systolic/diastolic pressures and flow or pressure waveforms. Blanco et al. [4] solved for resistance parameters in a detailed 1D network using Broyden’s method to match regional flow information. Using the adjoint method, Ismail et al. [5] proposed an optimization approach for estimating parameters in 3D FSI simulations. Alimohammadi et al. [6] presented an iterative minimization approach for parameter estimation in aortic dissection models. Xiao et al. [7] described a method for iteratively calibrating three-element Windkessel boundary conditions. Perdikaris and Karniadakis [8] demonstrated a Bayesian approach for discovering Windkessel parameters for 3D models, using an efficient global optimization (EGO) approach and two 1D surrogate models.

Previous approaches utilizing the Kalman filter include the work of DeVault et al. [9], who calibrated boundary condition parameters in a 1D model of the Circle of Willis using an Ensemble Kalman Filter (EnKF), incorporating velocity and pressure data. Similarly, Lal et al. [10] used the EnKF to determine Young’s modulus, together with boundary reflection and viscoelastic coefficients in 1D vessel networks. Pant et al. [11, 12] proposed a method or to determine boundary condition parameters for a 3D model by applying the Unscented Kalman filter (UKF) to a surrogate 0D model. Lombardi [13] applied the UKF to determine arterial stiffness and outflow boundary condition parameters in 1D vascular networks. Müller et al. [14] used the reduced order unscented Kalman filter (ROUKF) [15] to estimate total resistance, total compliance, and vessel wall properties in a 1D arterial network model, performing estimation steps in the frequency domain, and thus updating parameter estimates after each complete cardiac cycle. This approach requires minimal data, but does not independently adjust all model parameters. Similarly, Caiazzo et al. [16] used ROUKF with a 1D model to estimate arterial wall properties and terminal resistances.

It is striking that none of the above utilized Kalman filtering techniques in 3D; they instead work in 1D, or utilize a surrogate 0D or 1D model to generate parameter estimates for an associated 3D model. Broadly, all these methods are limited by the computational demand of their application to full-fidelity 3D models, inapplicability to arbitrary boundary condition designs, or both. In the present work, we demonstrate the use of the ROUKF for the estimation of three-element Windkessel model parameters, and of arterial wall stiffness, in subject-specific, 3D Navier–Stokes models of the aorta, and applied to the assimilation of data consisting of pressure waveforms, flow waveforms, and wall motion data. The key feature of ROUKF is that the uncertainty is confined to the parameters we wish to estimate, as opposed to the entire state space, making filtering of 3D PDE problems computationally tractable. A core advantage is that—unlike in many previous approaches—the high-fidelity forward model is used, without reliance on simplified surrogates. This ensures that the effect of complex, pathophysiological geometries can be retained, and is critical when spatial localisation of the observation data is important. We also introduce a ROUKF method augmented by constrained least squares (ROUKF-CLS), which enables filtering when the LPNs are more complex than the basic three-element Windkessel.

All the methods described in this paper are implemented as part of the CRIMSON (*Cardiovascular Integrated Modelling and Simulation*) software package [17], which comprises both a GUI for vessel segmentation from medical images, finite element mesh generation and boundary condition design and specification; and a stabilized, massively-parallel incompressible Navier–Stokes flowsolver. Of particular relevance is CRIMSON’s unique system for designing arbitrary LPN boundary condition circuits—the so-called *Netlist* boundary condition system—which integrates the ROUKF-CLS method introduced herein. The core ROUKF functionality is provided via integration with the Verdandi data assimilation library [18].

The outline of this paper is as follows: we first describe the methodology for data acquisition, followed by the formulation for the 3D FSI computational model and the ROUKF estimation algorithm. We then describe the augmented ROUKF-CLS method for filtering LPNs more complex than the three-element Windkessel. Finally, the functionality of these methods is demonstrated in a number of cases. The key results are a determination of Windkessel parameters in a subject-specific model of the whole aorta and main branches, and a demonstration of parameter recovery in a synthetic coronary LPN model using ROUKF-CLS.

## Methods

In this section, we present the novel algorithms and the data acquisition strategies for both synthetic and subject-specific data. In “Results” section, these data will be used to evaluate the efficacy of the algorithms for estimating LPN and wall material property parameters in a number of cases, including simplified tubes with either a Windkessel or a coronary-specific LPN structure, and multi-outlet Windkessel cases in a subject-specific aortic geometry.

### Data acquisition

#### Synthetic data

Synthetically-generated pressure, velocity and wall-motion data are used to validate the methods when we are in possession of the ground-truth, in a number of models. In some cases, our synthetic data generation uses idealised vascular geometries (tubes), and others use subject-specific arterial models. Each involves choosing specific values for the LPN or wall properties which we wish to ultimately estimate, imposing an inflow rate, and running a forward simulation, whilst acquiring the synthetic observation data on pressures, velocities or wall displacement, each at specific spatial locations. The precise workflow for each will be described with the results themselves, as they differ case-to-case. An example setup for one of these cases is shown in Fig. 2.

#### Subject-specific data

**Fig. 1:**
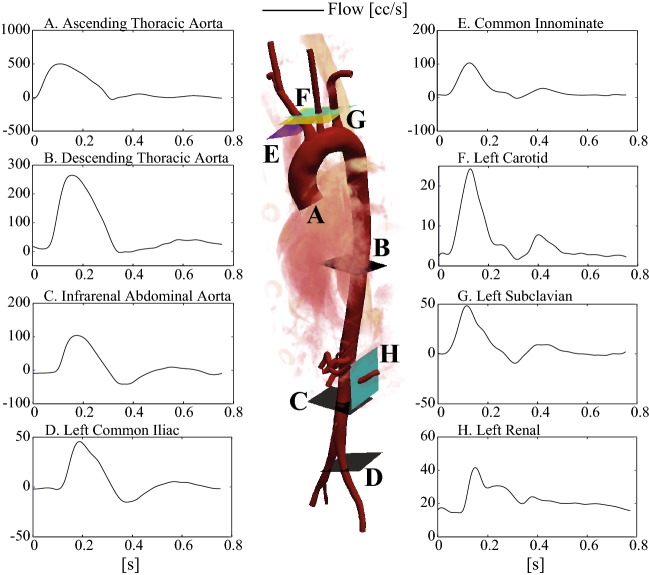
Vascular geometry, reconstructed from MRI data, showing the PC-MRI flow acquisition planes, and the corresponding flow waveforms

3D anatomical MRI was obtained from a 28-year-old male volunteer on a 1.5 T MR-system (Achieva, Philips Healthcare, the Netherlands) using cardiac triggering and respiratory navigation. From this, the aorta and surrounding major vessels were segmented and meshed, in preparation for simulation. Through-plane velocity data was acquired with high temporal and spatial resolution at three levels of the aorta (ascending, distal descending, and infra-renal) using 2D phase-contrast MRI (PC-MRI); the supra-aortic vessels; the left renal artery, and the left iliac artery. Figure 1 shows the segmented geometry, the PC-MRI acquisition locations and the corresponding flow waveforms. Time-resolved pressure waveforms were acquired using applanation tonometry (AtCor Medical SphygmoCor) in the left and right carotid artery while the subject was in a supine position. Ethical approval was obtained from St. Thomas’ Hospital Research Ethics Committee/South East London Research Ethics Committee (10/H0802/65). Details on the acquisition are provided in Appendix: MRI data acquisition and geometric segmentation and Pressure data acquisition.

### 3D blood flow formulation

We wish to use the ROUKF to determine parameters for an incompressible Navier-Stokes model of flow in a space-time vascular domain, $(\vec {x},t)\in \Omega ^{\mathrm{f}}\times (0,T)$, where $\Omega ^{\mathrm{f}}\subset {\mathbb {R}}^3$. The weak form of the FSI problem is as follows. Find the velocity $\vec {v}\in {\mathcal {S}}$ and pressure $p\in {\mathcal {P}}$ such that for all test functions $\vec {w}\in {\mathcal {W}}$ and $q\in {\mathcal {P}}$,

1 $$\begin{aligned}&\int _{\Omega ^{\mathrm{f}}} \{ \vec {w}\cdot (\rho _{\mathrm{f}}\vec {\dot{v}}+ \rho _{\mathrm{f}}\vec {v}\cdot \vec {\nabla }\, \vec {v}) + \vec {\nabla }\, \vec {w}: (-p\varvec{I}+{\varvec{\tau }}_{\mathrm{f}}) - \vec {\nabla }\, q\cdot \vec {v}\} d\vec {x} \nonumber \\&\quad + \int _{\Gamma _{\mathrm{in}}} q\vec {v}\cdot \vec {n}_{\mathrm{f}}da +\int _{\Gamma _{\mathrm{out}}} \{q\vec {v}\cdot \vec {n}_{\mathrm{f}}- \vec {w}\cdot \vec {h} \} da \nonumber \\&\quad +\int _{\Gamma _{\mathrm{w}}} \{ q\vec {v}\cdot \vec {n}_{\mathrm{f}}+ h \rho _{\mathrm{s}}\vec {w}\vec {\dot{v}}+ h \vec {\nabla }\, \vec {w}: {\varvec{\sigma }}_\mathrm{s}(\vec {u}) \}da = 0, \end{aligned}$$

where the solution and test function spaces are ${\mathcal {S}}= \{\vec {v}| \vec {v}(\cdot ,t)\,\in H^1(\Omega ^{\mathrm{f}}), t \in [0,T], \vec {v}(\cdot ,t) = \vec {g} \text { on } \Gamma _{\mathrm{in}}\}$, ${\mathcal {P}}= \{p| p(\cdot ,t)\,\in H^1(\Omega ^{\mathrm{f}}), t \in [0,T] \}$, and ${\mathcal {W}}= \{\vec {w}| \vec {w}(\cdot ,t)\,\in H^1(\Omega ^{\mathrm{f}}), t \in [0,T], \vec {w}(\cdot ,t) = 0 \text { on } \Gamma _{\mathrm{in}}\}$. Here, $H^1(\Omega ^{\mathrm{f}})$ is the space of real-valued functions on $\Omega ^{\mathrm{f}}$ whose values and first derivatives are square-integrable.

Here, $\vec {v}(\vec {x},t)$ denotes the velocity field, $p(\vec {x},t)$ the pressure field, ${\varvec{\tau }}_{\mathrm{f}}= \mu \{ \vec {\nabla }\, \vec {v}+ ( \vec {\nabla }\, \vec {v})^\text {T}\}$ is the viscous stress tensor, $\mu$ is the dynamic viscosity of the blood, and $\rho _{\mathrm{f}}$ is its density and $\vec {u}$ the vessel wall displacement. The boundary conditions for velocity and traction are given by:

2 $$\begin{aligned} \begin{array}{rcllcl} \vec {v}(\vec {x},t) &{} = &{} \vec {g}(\vec {x},t) &{}(\vec {x},t) &{} \in &{} \Gamma _{\mathrm{in}}\times (0,T), \\ (-p\varvec{I}+{\varvec{\tau }}_{\mathrm{f}})\vec {n}_{\mathrm{f}}&{} = &{} \vec {h}(\vec {v},p,\vec {x},t) &{}(\vec {x},t) &{} \in &{} \Gamma _{\mathrm{out}}\times (0,T), \end{array} \end{aligned}$$

where $\vec {g}(\vec {x},t)$ is a prescribed velocity on the inflow boundary $\Gamma _{\mathrm{in}}$, and $\vec {h}$ is the traction on the boundary $\Gamma _{\mathrm{out}}$. $\vec {n}_{\mathrm{f}}$ denotes the outward-pointing boundary unit normal vector. On each connected component of the boundary $\Gamma _{\mathrm{out}}$, $\vec {h}$ is determined by an LPN model of the downstream vasculature. This is implicitly coupled to the 3D domain via the Coupled Multidomain method of Vignon-Clementel et al. [19, 20]. $\Gamma _{\mathrm{w}}$ denotes the interface between the blood and the vessel wall, which is represented as a thin linear membrane with thickness *h* and Cauchy stress ${\varvec{\sigma }}_\mathrm{s}(\vec {u})$ using the Coupled Momentum Method of Figueroa et al. [21]. The displacement $\vec {u}$ is calculated from the nodal velocities and accelerations for each time step using a Newmark integration scheme. The enhanced membrane Cauchy stress tensor ${\varvec{\sigma }}_\mathrm{s}$ is given as a function of a tensor ${\varvec{\tilde{K}}}$ of material parameters stiffness $E$, Poisson’s ratio $\nu$, and transverse shear factor $k$; a tensor ${\varvec{P}}$ describing the pre-stress of the wall, and the infinitesimal strain tensor ${\varvec{\epsilon }}(\vec {u})$; ${\varvec{\sigma }}_\mathrm{s}= \tilde{\varvec{K}}(E,\nu ,k) : {\varvec{\epsilon }}(\vec {u}) + {\varvec{P}}$. The pre-stress tensor can be specified using a variety of methods [22–24].

#### Boundary conditions: three-element Windkessel LPN

**Fig. 2:**
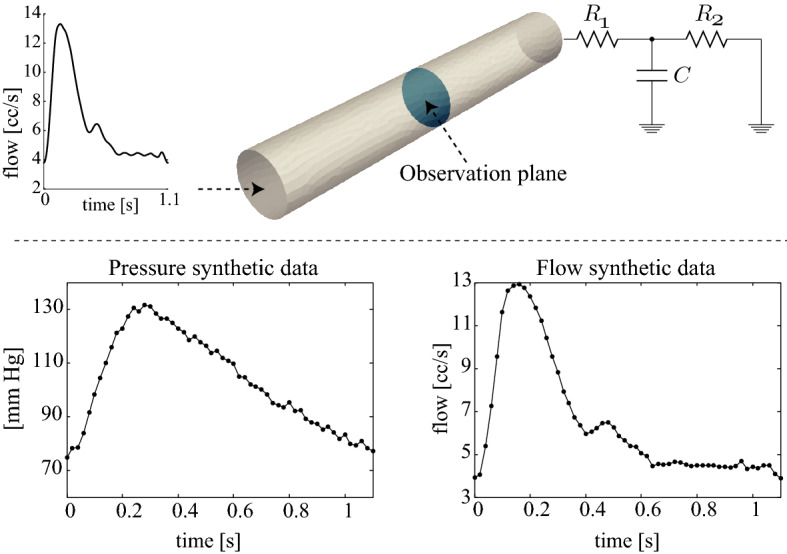
Top: problem setup for an idealized carotid artery. The plane where the volumetric flow and cross section-averaged pressure are observed is denoted in blue. The inflow velocities are prescribed using a typical carotid flow waveform and a parabolic velocity profile. The outflow face is coupled to a three-element Windkessel. Bottom: flow and pressure synthetic data (with added Gaussian white noise) created from the mid-vessel observation site during the forward simulation

The most commonly used LPN boundary condition is the three-element Windkessel model. Its parameters are a proximal resistance $R_{1}$, a distal resistance $R_{2}$, and a compliance *C*. Its structure can be seen in Fig. 2. At the interface $\Gamma _{\mathrm{out}}$ with the 3D domain, pressure, *P*(*t*) and flow, *Q*(*t*), in the LPN are related by the ordinary differential equation:

3 $$\begin{aligned} Q \left( 1 + \frac{R_1}{R_2} \right) + C R_1 \frac{d Q}{d t} = \frac{P}{R_2} + C \frac{d P}{d t}. \end{aligned}$$

From this, together with a choice of implicit temporal discretization scheme for Eq. 3, and using a boundary condition time-step $\Delta t_{BC}$, the expression:

4 $$\begin{aligned} -\int _{\Gamma _{out}^{i}} \vec {w}\cdot \vec {h} da =&\int _{\Gamma _{out}^{i}} \vec {w}\cdot \vec {n}_{\mathrm{f}}\bigg \{ \left( R_{1}+\frac{R_{2}}{1+C R_{2}/\Delta t_{BC}}\right) \nonumber \\&\cdot \left( \int _{\Gamma _{out}^{i}} \vec {v}\cdot \vec {n}_{\mathrm{f}}da\right) \nonumber \\&+ \frac{(P-R_{1}Q)C R_{2}}{\Delta t_{BC} + C R_{2}} \bigg \} da \end{aligned}$$

can be derived. Here, $\Gamma _{out}^{i}\subset \Gamma _{\mathrm{out}}$ is the *i*-th outlet, to which this LPN is coupled, and $\Delta t_{BC}$ is dependent upon the time-stepping scheme chosen for the PDE; see “Space–time discretization” section. This can be inserted into Eq. 1 to couple the LPN to the 3D domain.

#### Boundary conditions: arbitrary *Netlist* LPNs

To couple arbitrary LPNs to the 3D domain without manually deriving an ordinary differential equation on pressure *P*(*t*) and flow *Q*(*t*) equivalent to Eq. 3 for each, we make use of our *Netlist* boundary condition system [17]. This permits the design and coupling of LPNs representing more complex vascular beds; for example, the unique features of the coronary circulation [25], or of the heart [26], or even of a closed-loop circulatory system of patients with single-ventricle physiology [27]. Briefly described, the Netlist system takes a user-defined arbitrary LPN structure, designed using the CRIMSON GUI [17], and converts it into a matrix system of equtions for the LPN state, from which the time-dependent values *effective resistance* ${\tilde{R}}(t)$ and *pressure shift* ${\tilde{S}}(t)$ can be deduced. The term in $\vec {h}$ in Eq. 1 can then be expressed via

$$\begin{aligned} -\int _{\Gamma _{out}^{i}} \vec {w}\cdot \vec {h} da = \int _{\Gamma _{out}^{i}} \vec {w}\cdot \vec {n}_{\mathrm{f}}\bigg \{ {\tilde{R}} \left( \int _{\Gamma _{out}^{i}} \vec {v}\cdot \vec {n}_{\mathrm{f}}da\right) + {\tilde{S}} \bigg \} da \end{aligned}$$

The details of the determination of the coefficients ${\tilde{R}}(t)$ and ${\tilde{S}}(t)$ are beyond the scope of the present work, and will be presented in a separate publication.

#### Space–time discretization

Equation 1 is spatially discretized using a stabilized finite element formulation with equal-order interpolation spaces for velocity and pressure on a tetrahedral volumetric mesh [28–31]. Time discretization is achieved using the generalized $\alpha$ method [32, 33].

### Parameter estimation method

#### Definition of the forward model

Consider the spatially and temporally discretized nonlinear system of equations arising from the finite element formulation of the blood flow problem at time step *k*,

5 $$\begin{aligned} X_k&= [v_k,p_k,\dot{v}_k,u_k, l_{k}]^\text {T}, \nonumber \\ X_0&= X^0, \nonumber \\ \theta _0&= \theta ^0 + \xi _\theta , \nonumber \\ X_k&= A(X_{k-1},\theta _k). \end{aligned}$$

Here, $v_k,\dot{v}_k,p_k$, and $u_k$ are vectors containing the finite element basis function solution weights for the velocity, acceleration, pressure, and wall displacement fields, respectively; and $l_{k}$ is a vector of boundary condition internal state variables. Together they form the model state, $X_k$, at time step *k*. Application of the operator *A* updates the FSI model state by one time step, from $X_{k-1}$ to $X_{k}$, given the current parameter set $\theta _{k}$, which contains the model parameters to be estimated. $X^0$ and $\theta ^0$ are *a priori* initial values, and $\xi _\theta$ represents parameter uncertainty. The combined vector $\{X_k,\theta _k\}$ is called the augmented state.

In the case where only Windkessel parameters are to be estimated, the components of $\theta _k$ can be written:

6 $$\begin{aligned} \theta _k= & {} [ {\tilde{r}}_1^{(1)},{\tilde{r}}_1^{(2)},\ldots ,{\tilde{r}}_1^{(n_\text {out})},\nonumber \\&{\tilde{r}}_2^{(1)},{\tilde{r}}_2^{(2)},\ldots ,{\tilde{r}}_2^{(n_\text {out})}, \nonumber \\&{\tilde{c}}^{(1)},{\tilde{c}}^{(2)},\ldots ,{\tilde{c}}^{(n_\text {out})}]_k^\text {T}, \nonumber \\ R_1^{(j)}= & {} 2^{{\tilde{r}}_1^{(j)}}, \ \ R_2^{(j)} = 2^{{\tilde{r}}_2^{(j)}}, \ \ C^{(j)} = 2^{{\tilde{c}}^{(j)}} \end{aligned}$$

where $R_1^{(j)}$, $R_2^{(j)}$, and $C^{(j)}$ are the proximal resistance, distal resistance, and compliance at the *j*-th outlet, respectively. When there are $n_\text {out}$ Windkessel models for which we wish to estimate parameters, the total number of parameters is $N= 3n_{\text {out}}.$ Re-parameterization in terms of powers of two in Eq. 6 prevents estimation of negative values of resistance and compliance.

#### Estimation algorithm—ROUKF for three-element Windkessel LPNs and vessel stiffness

The ROUKF is based on the Unscented Kalman Filter (UKF) formulation of Julier et al. [34, 35]. This extension of the Kalman filter to nonlinear models relies on a deterministic, discrete sampling of the estimation error probability distribution with a set of particles or *sigma points*; these are perturbations of the model parameters around the current best estimate of their values. The ROUKF is designed for parameter estimation in large dynamical systems where the number of parameters is comparatively smaller than the number of state variables, and where it is assumed that the uncertainty in the system can be solely attributed to the parameters. Whereas the classical Kalman filter and its variants call for computations involving a full estimation error covariance matrix, $P$, with the same dimension as the augmented state-space (state variables plus the uncertain parameters) at every time step, the key idea behind the ROUKF is restricting the uncertainty to a small part of the augmented state space (i.e. the parameters). In this case, $P$ is factorized as $P= L{U}^{-1} {L^{\text {T}}}$, where $U$ is a $N$-by-$N$ square matrix, $L$ is a *M*-by-$N$ matrix, $N$ is the number of uncertain parameters and *M* the total number of state variables plus uncertain parameters. This allows the uncertain parameter covariances to be tracked in the small matrix $U$, and makes computations involving the estimation error covariance matrix, $P$, computationally tractable for large problems.

**Fig. 3:**
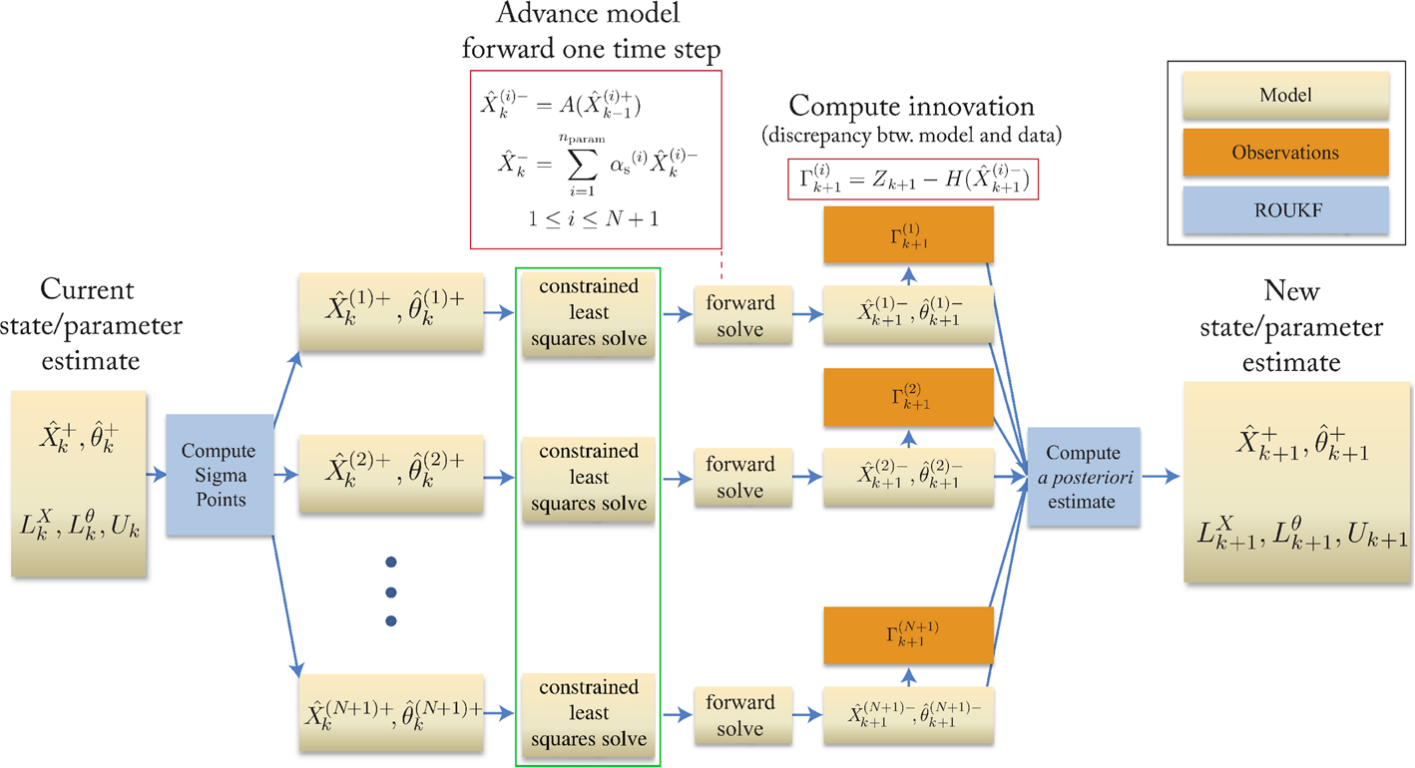
Diagram of the estimation procedure during a single time step, showing the interaction between the FSI model and the filtering algorithm. The green box identifies the constrained least squares step, described in “Estimation algorithm—ROUKF-CLS for arbitrary LPNs” section, which is only part of the ROUKF-CLS algorithm (not of the basic ROUKF procedure)

Let $\hat{X}_k, \hat{\theta }_k$ be the state and parameters estimates at time step *k*, respectively, and let $L_{k}$ be $L$ at time step *k*. For convenience, we further define $L^{X}_k$ and $L^\theta _k$, which are matrices formed from the rows of $L_k$ corresponding to the state variables and model parameters, respectively. The estimation procedure is summarized next and depicted graphically in Fig. 3. Note that the step in the green box in Fig. 3 is not part of this basic ROUKF algorithm (for basic ROUKF, it is simply omitted, passing directly to the next step), and will be formally introduced in “Estimation algorithm—ROUKF-CLS for arbitrary LPNs” section.

1. Precompute the $N+1$ simplex sigma point direction vectors, $\sigma ^{N}_{(i)}$ [36]. The details of the procedure are described in Appendix: Sigma point generation.
2. Initialize the error covariance factors,
  7 $$\begin{aligned} L^{X}_0&= \left[ \begin{array}{l} 0_{(M-N)\times N} \end{array} \right] \nonumber \\ L^\theta _0&= \left[ \begin{array}{l} I_{N} \end{array} \right] \nonumber \\ U_0&= \left[ \begin{array}{llll} 1/s_1 &{} &{} \\ &{} 1/s_2 &{} \\ &{} &{} \ddots \\ &{} &{} &{} 1/s_{N} \end{array} \right] . \end{aligned}$$
  Here, $U_0$ is assumed to be diagonal, $0_{(M-N)\times N}$ is a $(M-N) \times N$ matrix of zeros, and $I_{N}$ is the $N\times N$ identity matrix. The values $s_1, s_2, \ldots , s_{N}$ represent the confidence in the initial guess for the parameters.
3. Using the sigma point direction vectors, create the set of $N+1$ sigma points around the current estimate at time step *k*:
  8 $$\begin{aligned} \hat{X}_{k}^{(i)+}&= \hat{X}_{k}^{+} + L^{X}_{k}\sqrt{(U_{k})^{-1}}\sigma ^N_{(i)} \nonumber \\ \hat{\theta }_{k}^{(i)+}&= \hat{\theta }_{k}^{+} + L^\theta _{k}\sqrt{(U_{k})^{-1}}\sigma ^N_{(i)} \nonumber \\ 1&\le i \le N+ 1, \end{aligned}$$
  where the square root may be computed using Cholesky factorization. Each sigma point consists of state and parameter variables of the model,
  9 $$\begin{aligned} \hat{X}_{k}^{(i)+} = [v_{k_{(i)}},p_{k_{(i)}},\dot{v}_{k_{(i)}}, u_{k_{(i)}},l_{k_{(i)}}]^\text {T}, \ \ \hat{\theta }_{k}^{(i)+}. \end{aligned}$$
  Here, $l_{k}$ represents the internal state (zero-dimensional pressures and flows) in the boundary condition LPNs.
4. Propagate each sigma point forward by one time step, using the finite element formulation described in “3D blood flow formulation” section. Each one of these independently-propagating states is called a simulation *particle*.
  Upon completion of the $N+1$ forward solves, the updated sigma points, indexed by *i*, are given by:
  10 $$\begin{aligned} \hat{X}_{k+1}^{(i)-}= & {} [v_{k+1_{(i)}},p_{k+1_{(i)}},\dot{v}_{k+1_{(i)}}, u_{k+1_{(i)}},l_{k+1_{(i)}}]^\text {T}, \nonumber \\&\hat{\theta }_{k+1}^{(i)-} \ \ 1 \le i \le N+ 1. \end{aligned}$$
5. Compute the ensemble mean, to obtain the *a priori* estimate:
  11 $$\begin{aligned} \hat{X}_{k+1}^{-}&= \sum _{i=1}^{N} {\alpha _{\mathrm{s}}^{(i)}} \hat{X}_{k+1}^{(i)-} \nonumber \\ \hat{\theta }_{k+1}^{-}&= \sum _{i=1}^{N} {\alpha _{\mathrm{s}}^{(i)}} \hat{\theta }_{k+1}^{(i)-}, \end{aligned}$$
  where the $\alpha _{\mathrm{s}}^{(i)}$ are the sigma point weights (see Appendix: Sigma point generation).
6. Compute the updated innovation, $\Gamma _{k+1}$, using the measurements $Z_{k+1}$ and the state component of the sigma points, $\hat{X}_{k+1}^{(i)-}$:
  12 $$\begin{aligned} \Gamma _{k+1}^{(i)}&= Z_{k+1}-H(\hat{X}_{k+1}^{(i)-})  1 \le i \le N+ 1. \end{aligned}$$
  Here, $Z_{k+1}$ is the recorded patient data—for example, the instantaneous volumetric blood flow through a vessel of interest at the current time step—and $H$ is the operator which mimics this measurement in the model—such as the instantaneous flow through a cross-sectional plane.
7. Compute the updated estimation error covariance factors:
  13 $$\begin{aligned} L^{X}_{k+1}&= [\hat{X}_{k+1}^{(*)-}] D_{\alpha }[\sigma _{(*)}]^\text {T}\nonumber \\ L^\theta _{k+1}&= [\hat{\theta }_{k+1}^{(*)-}] D_{\alpha }[\sigma _{(*)}]^\text {T}\nonumber \\ \{HL\}_{k+1}&= [\Gamma _{k+1}^{(*)}] D_{\alpha }[\sigma _{(*)}]^\text {T}\nonumber \\ U_{k+1}&= P_\alpha + \{HL\}^\text {T}_{k+1} W^{-1}_{k+1} \{HL\}_{k+1} \end{aligned}$$
  where $[\hat{X}_{k+1}^{(*)-}]$, $[\hat{\theta }_{k+1}^{(*)-}]$, $[\Gamma _{k+1}^{(*)}]$, and $[\sigma _{(*)}]$ are matrices whose *i*-th columns are $\hat{X}_{k+1}^{(i)-}$, $\hat{\theta }_{k+1}^{(i)-}\,\Gamma _{k+1}^{(i)}$, and $\sigma ^N_{(i)}$, respectively. The diagonal matrix $D_{\alpha }$ stores the weights $\alpha _{\mathrm{s}}^{(i)}$ associated to each sigma-point (see Appendix: Sigma point generation) and $W_{k+1}$ is the measurement error covariance matrix described in Appendix: Measurement error covariance matrix.
8. Compute the updated *a posteriori* estimate:
  14 $$\begin{aligned} \hat{X}_{k+1}^{+} =&\hat{X}_{k+1}^{-} \nonumber \\&- L^{X}_{k+1}U_{k+1}^{-1}\{HL\}_{k+1}^\text {T}W^{-1}_{k+1} \sum _{i=1}^{N}\alpha _{\mathrm{s}}^{(i)} \Gamma _{k+1}^{(i)} \nonumber \\ \hat{\theta }_{k+1}^{+}&= \hat{\theta }_{k+1}^{-} \nonumber \\&- L^\theta _{k+1}U_{k+1}^{-1}\{HL\}_{k+1}^\text {T}W^{-1}_{k+1} \sum _{i=1}^{N}\alpha _{\mathrm{s}}^{(i)} \Gamma _{k+1}^{(i)}. \end{aligned}$$
  This provides the updated estimate of the state of System 5 given the data $Z$; in particular, this includes updated best-estimates of the parameters $\theta$ that we are trying to determine.
9. Repeat from step 3 after incrementing the time index: $k+1 \rightarrow k$.

In this work, this ROUKF method is used to estimate vessel stiffness and three-element Windkessel model parameters.

#### Estimation algorithm—ROUKF-CLS for arbitrary LPNs

We now motivate and describe the modification to the above ROUKF method required to obtain the ROUKF-CLS algorithm. ROUKF-CLS is required for parameter estimation in LPNs more complex than the three-element Windkessel model. The reason is that general, time-discretized LPNs have too many time-dependent pressure and flow “history” states, due to the appearance of time derivatives in the LPN equations. For any time step index *k*, the value of these states is a function of the historical behavior of the model, and of the value of the LPN parameters. When a particle is generated, this state at step *k* is inconsistent with the new parameters, so it must be updated before advancing to $k+1$. To achieve this, an additional step is taken after particle generation, between Steps 3 and 4 in “Estimation algorithm—ROUKF for three-element Windkessel LPNs and vessel stiffness” section; we refer to this as the *consistency step*. Specifically, the 3D-to-LPN interface flow rate and interface pressure at time-step *k* are imposed upon the LPN, and consistent internal state variables are computed. This approach is similar in spirit to those utilizing projection-based constraint of the state onto some subspace [37, 38]. This additional step is shown in the green box in Fig. 3.

We now describe the consistency step of ROUKF-CLS. Let the time-discretized linear system for the LPN be given by:

15 $$\begin{aligned} L_{LPN} {\mathbf {x}} = {\mathbf {b}}, \end{aligned}$$

where $L_{LPN}$ is a matrix containing the standard equations for resistors, capacitors, etc., representing the LPN; ${\mathbf {x}}$ is the vector of unknown pressure and flow variables for time-step *k* inside the LPN; and ${\mathbf {b}}$ contains any necessary state from time-step $k-1$ (i.e. from $l_{k-1}$; see Eq. 9), together with the 3D domain flow rate at step *k*. This system is square.^1^ To enforce consistency with the 3D domain, we add a row to this matrix equation, imposing the *a posteriori* 3D interface pressure from time step *k* upon the LPN, obtaining the overdetermined system,

16 $$\begin{aligned} L_{LPN}^{over} {\mathbf {x}} = {\mathbf {b}}^{over}. \end{aligned}$$

Next, we create a second linear system as follows. We remove all rows enforcing the state $l_{k-1}$ at time-step $k-1$ from Eq. 16, obtaining

17 $$\begin{aligned} L_{LPN}^{under} {\mathbf {z}} = {\mathbf {d}}, \end{aligned}$$

which amounts to deconstraining of the problem from a historical state which was inconsistent with the new parameters. If there were more than one such row, this system is underdetermined.^2^

Since the filter-induced changes in the LPN parameters for each time-step are small, we assert now that while the state at step $k-1$ has been deconstrained, we do not want to deviate far from it. We also know that the solution must exactly satisfy the pressure-flow relationships across each LPN component at time *k*. Taking these concepts together, we obtain a constrained least squares problem:

18 $$\begin{aligned}&\mathrm {minimize}~\left\| L_{LPN}^{over} {\mathbf {x}} - {\mathbf {b}}^{over}\right\| , \end{aligned}$$

19 $$\begin{aligned}&\mathrm {~subject~to~the~constraint~} L_{LPN}^{under} {\mathbf {z}} = {\mathbf {d}}, \end{aligned}$$

which can be formulated as: find ${\mathbf {x}}$ such that,

20 $$\begin{aligned} \left( \begin{array}{cc} {L_{LPN}^{over}L_{LPN}^{over}}^{T} &{} {L_{LPN}^{under}}^{T} \\ {L_{LPN}^{under}} &{} {\mathbf {0}} \\ \end{array} \right) \left( \begin{array}{c} {\mathbf {x}} \\ {\mathbf {z}} \\ \end{array} \right) = \left( \begin{array}{c} {L_{LPN}^{over}}^{T}{\mathbf {b}}^{over} \\ {\mathbf {d}} \\ \end{array} \right) . \end{aligned}$$

We solve this system each time a particle is generated, obtaining a particle-specific consistent internal state $l_{k}$ for step *k*, ready for propagation to the $\textit{a priori}$ state at $k+1$, in Step 4 of “Estimation algorithm—ROUKF for three-element Windkessel LPNs and vessel stiffness” section. We refer to the ROUKF algorithm, augmented by the consistency step involving Eq. 20, as ROUKF-CLS.

#### Model observations and real-world data

The measurement term $Z_k$ at time step *k* is a vector of clinically-recorded flow and pressure waveforms. The innovation term $Z_k-H(X_k)$ in Eq. 12 is thus the discrepancy between the model-predicted and real-world values of blood pressure and flow. The observation operator *H* extracts data from the model. To mimic the patient data we wish to assimilate, we define it as:

$$\begin{aligned} H(X_k)&= [Q_1,Q_2,\ldots ,Q_{n_\text {obs-Q}},P_1,P_2,\ldots ,P_{n_\text {obs-P}}]^\text {T}_k, \end{aligned}$$

where volumetric flow rate and spatially-averaged pressure are respectively defined by:

21 $$\begin{aligned} Q_i&= \int _{\Pi _i} \vec {v}^h(\vec {x},t)\cdot \vec {n}_{i} da, \nonumber \\ P_i&= \frac{1}{\text {area}(\Pi _{i})}\int _{\Pi _i} p^h(\vec {x},t) da, \end{aligned}$$

where $\Pi _{i}$ is the intersection of a plane with the 3D domain. Here, $\vec {n}_{i}$ is a choice of unit normal to $\Pi _{i}$. Compare the PC-MRI planes shown in Fig. 1; typically, these planes mimic the location of the PC-MRI measurement planes used to record the patient data.

**Fig. 4:**
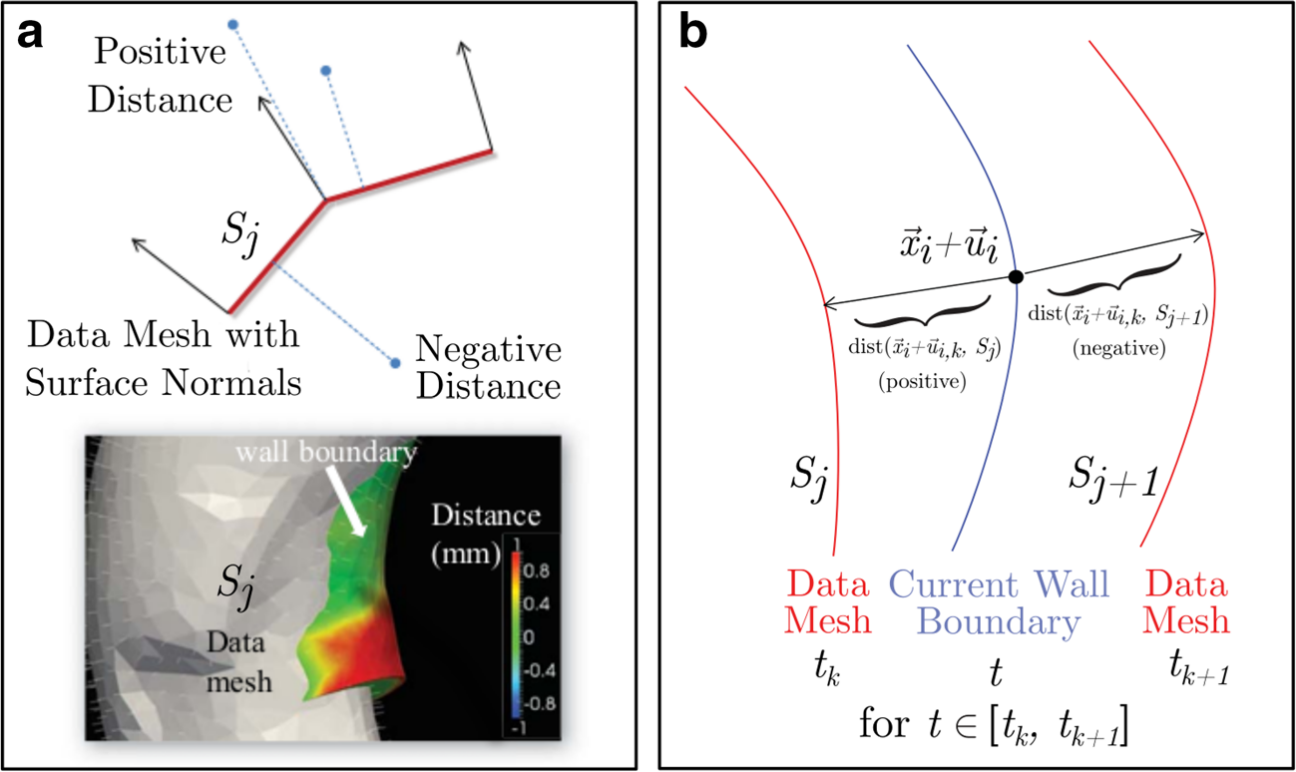
Signed wall distance metric for wall motion observations. **a** The distance metric is the Euclidean distance between a point and the surface, and is positive in the outward-pointing unit normal direction, and negative otherwise. A distance map between points on the deformed wall boundary and the *j*-th data surface $S_{j}$ is shown. **b** The signed metric is used to compute an interpolated distance between consecutive times where wall position data $S_{j}$ are available. Note that the blue curve represents the current wall boundary; the “interpolated” surface $S_{t}$ between $S_{j}$ and $S_{j+1}$ is never constructed, and is not shown

When the available data includes wall motion, the definition of the innovation term in Eq. 12 can be made by means of a ‘distance’ operator between the wall position in the simulation and in the data. Suppose the data consists of a time-dependent series of *M* surfaces $S_{j}$, defining the vessel wall at times $t_{j}$, $j\in \left\{ 1,2,\dots , M\right\}$. Following [39], we define $dist(\vec {x}, S_{j})$ to be the signed Euclidean distance between $\vec {x}$ and the closest point to $\vec {x}$ on $S_{j}$. The sign is negative if $\vec {x}$ lies inside the volume delimited by $S_{j}$, and positive otherwise, see Panel A of Fig. 4. When surfaces $S_{j}$ are not available for every time-step of the simulation, we interpolate between them. We define $a_{j}(t)$ to be a linear function of *t* such that $a_{j}(t_{j})=0$ and $a_{j}(t_{j+1})=1$, and define the distance to the interpolated surface $S_{t}$ at time $t\in [t_{j}, t_{j+1}]$ by:

22 $$\begin{aligned} D(\vec {x}, S_{t})=a_{j}(t)dist(\vec {x}, S_{j}) + (1-a_{j}(t))dist(\vec {x}, S_{j+1}). \end{aligned}$$

For a distance observation at the *i*-th wall node $\vec {x}_{i}$, and its displacement $\vec {u}_{i,k}$ at time $t_{k}\in [t_{j}, t_{j+1}]$ for some *j* (Fig. 4b), we directly define the innovation as:

23 $$\begin{aligned} Z_{k}-H(X_k)=&a_{j}(t_{k})dist(\vec {x}_{i}+\vec {u}_{i,k}, S_{j}) \nonumber \\&+(1-a_{j}(t_{k}))dist(\vec {x}_{i} + \vec {u}_{i,k}, S_{j+1}). \end{aligned}$$

## Results

We present numerical results in the following cases: in “Idealized carotid artery with synthetic data” section, ROUKF Windkessel parameter estimation in an idealized carotid artery with synthetic data; in “Subject-specific aorta with synthetic data” and “Subject-specific aorta with PC-MRI and applanation tonometry data” sections, ROUKF Windkessel parameter estimation in a subject-specific aorta, with cases involving both synthetic and PC-MRI and applanation tonometry data; in “Reduced order Unscented Kalman Filter with constrained least squares (ROUKF-CLS)” section, the performance of the ROUKF-CLS is explored, first through a consistency test between ROUKF and ROUKF-CLS Windkessel parameter estimation (“ROUKF-CLS verification against ROUKF using a Windkessel model” section); and then in a case of coronary LPN parameter estimation, for which ROUKF is unsuitable (“ROUKF-CLS applied to a coronary LPN model” section).

### Idealized carotid artery with synthetic data

#### Estimation of Windkessel parameters from pressure and flow

A straight, deformable vessel with a prescribed inflow at one end, and a Windkessel model at the other was studied, as shown in Fig. 2. The inflow velocity was chosen to represent a typical carotid flow waveform, mapped to a parabolic velocity profile. The stiffness *E* of the vessel wall was 0.7 MPa and the thickness *h* was chosen to be 0.3 mm.

*Forward problem* Synthetic flow and pressure waveforms were generated using a simulation with known Windkessel parameters, run with a time-step of 1 ms, until cycle-to-cycle periodicity was achieved. During the final cardiac cycle, volumetric flow and cross-sectional averaged pressure waveforms were obtained at the vessel midplane (see Fig. 2) with a time-spacing of 20 ms (50 Hz sampling rate). Gaussian white noise was added to the waveforms with a signal-to-noise ratio (SNR) of 40 dB.

*Estimation problem* Six different estimation problems, A–F, were studied. In cases A, B and C, a single parameter of the Windkessel model shown in Fig. 2 was estimated—*C*, $R_2$, and $R_1$ respectively. In case D, *C* and $R_{2}$ were estimated, in case E, $R_{1}$ and $R_{2}$ were estimated, and in case F, all three parameters were estimated. In all cases, the initial estimation covariances $s_{i}$ were set to 0.2 (Eq. 7), and the measurement error covariances for the pressure and the flow data were set to $w_P^{(1)}=12.3^2~(\mathrm {mmHg})^{2}$ and $w_Q^{(1)}=1.21^{2}~(\mathrm {cc}/\mathrm {s})^{2}$ (Appendix: Measurement error covariance matrix).

**Fig. 5:**
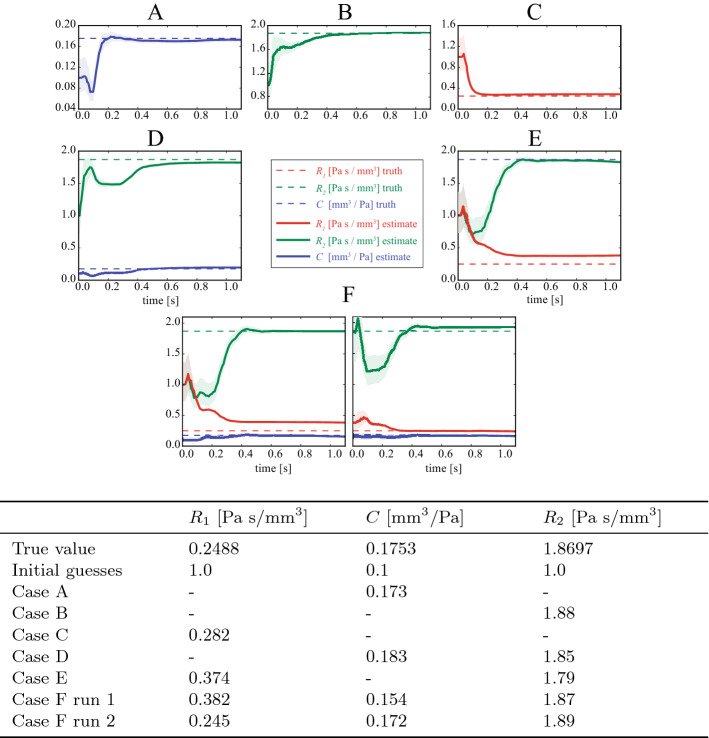
Evolution of the parameters in the idealized carotid example with synthetic data. In cases **A**–**C** (top) only a single parameter was estimated. In cases **D**, **E** (middle), two parameters were simultaneously estimated. In case **F** (bottom) all three parameters were simultaneously estimated. The true values, initial guesses, and final estimated values for the parameters are shown in the table. The shaded regions depict plus/minus the estimation error standard deviation

Figure 5 shows the evolution of the estimated parameters from their initial guesses in each of the six cases. We observed that in cases A–D, convergence to the true value was achieved after one cardiac cycle (1.1 s). In cases E and F, the estimation run does not fully recover the true parameters. For case F (both of the lower-most two panels of Fig. 5), the right plot shows that restarting the estimation using the parameter values reached by the end of the initial (left plot) run results in better convergence, highlighting the importance of good initial guesses, as well as indicating a strategy for recovering when those guesses are poor. Note that in all cases shown, $R_{1}$ predominantly changes during the early part of the cycle, which corresponds to systole. This is because $R_{1}$ has the greatest impact on the results during this period. During diastole, $R_{2}$ and *C* are more important. In both cases E and F, $R_{1}$ does not change sufficiently during the first systole, so a second cycle is required (shown by the two panels for Case F).

#### Simultaneous estimation of wall stiffness and Windkessel compliance

**Fig. 6:**
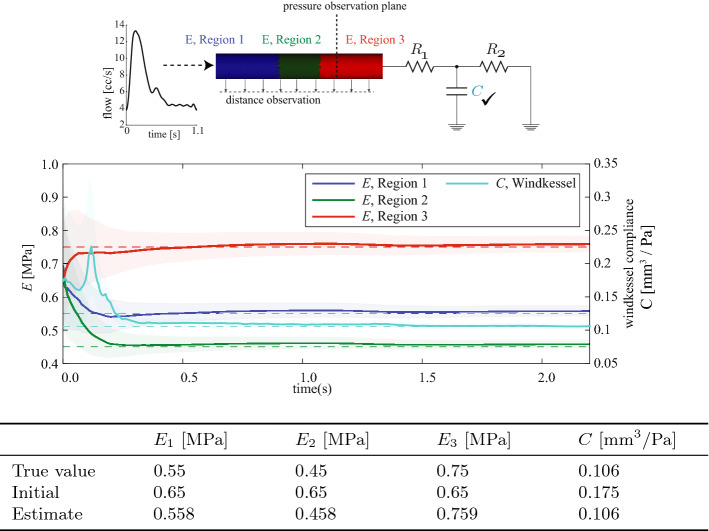
Simultaneous estimation of wall stiffnesses and Windkessel compliance. Top: the simulation set-up. The vessel surface is divided into three regions, each with a different value of stiffness, *E*. The inflow rate is given by a prescribed waveform, and a three-element Windkessel model is coupled at the outlet. The three values of *E* and the Windkessel compliance, *C*, will be estimated. Middle: evolution of the parameters during estimation. Solid lines denote the estimates; dashed lines indicate their true values. Shaded regions depict the estimation standard deviation. Bottom: the results are summarized

We demonstrate simultaneous estimation of both the Windkessel compliance *C*, and wall stiffness, *E*. The total compliance of the vessel is determined by the combined effect of *E* and *C*, and it affects the pulse pressure amplitude. Conversely, the wall stiffness informs the wall deformation. To capture vessel stiffness and Windkessel compliance, we employ two observations (see Fig. 6): a cross-sectional averaged pressure; and a distance observation using wall motion data from the forward FSI simulation, implicitly defined by Eq. 23 [39–41].

*Forward problem* The cylindrical vessel shown in Fig. 6 was divided into three regions, with differing values of stiffness *E* in each, and a simulation was run until periodicity was achieved, using $C = 1.06\times 10^{-5}\,\hbox {cm}^5/\hbox {dyne} = 0.106\,\hbox {mm}^3/\hbox {Pa}$. From the final cardiac cycle, wall deformation observations composed of a series of twenty-four wall surfaces was extracted. Then, pressure data was obtained at the observation plane at 100 Hz, and subsequently adding Gaussian white noise at 30 dB SNR.

*Estimation problem* We employed $n_{w}$ nodal distance observations for each of the twenty-four wall surfaces, and one pressure observation. Initial guesses for the parameters were set to $E=0.65$ MPa (uniformly down the length of the vessel) and $C = 0.175\,\hbox {mm}^3$/Pa. Initial estimation error covariance values were $s_i = 0.5$. The observation covariance matrix $W_k$ was block-diagonal, with a diagonal entry $w_P=(12.3~\mathrm {mmHg})^2$ for the pressure variance, and a $n_{w}\times n_{w}$ block $(w^{-1}M_{\Gamma _{\mathrm{w}}})^{-1}$ for the wall node covariances, where $w = (0.1\hbox { mm})^2$ and $M_{\Gamma _{\mathrm{w}}}$ is a normalized mass matrix associated with the wall boundary [41].

The results of the sequential parameter estimation simulation are summarized in Fig. 6, demonstrating accurate recovery of both regional stiffness *E* and Windkessel *C* after two cardiac cycles. Of note, the region with the lowest stiffness $E = 0.45$ in the center of the vessel showed the smallest standard deviation in the the estimation error (green shaded region). This is likely due to this region having the largest wall displacement (and thus the largest signal).

### Subject-specific aorta with synthetic data

**Fig. 7:**
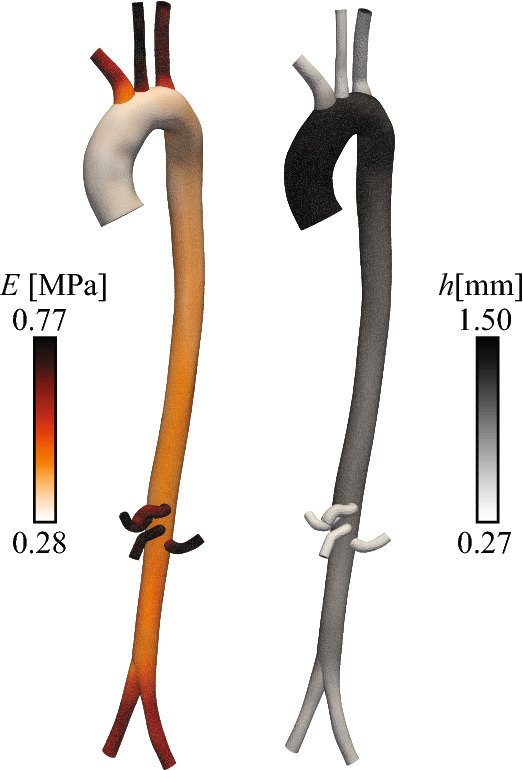
Spatial distribution of aortic wall properties. Left: stiffness, *E*. Right: wall thickness, *h*

We now consider a subject-specific aorta and its main branches, obtained as described in “Data acquisition” section. The geometric model has one inlet and nine outlets; we aim to recover $R_1$, $R_2$ and *C* at all nine outlets. Inlet velocities were prescribed using the ascending aortic PC-MRI data. Wall thickness *h* was set to be 10% of the local vessel radius. Vessel stiffness *E* was specified by an empirical formula relating pulse wave velocity to local vessel radius [42], scaled uniformly to match the subject-specific wave speed measured from the four flow waveforms in the aorta and iliac artery (see Fig. 1) [43]. The resulting maps of vessel wall properties are shown in Fig. 7. Simulations were performed using a finite element mesh comprising $\sim$ 220k linear tetrahedral elements, and a time-step of 0.25 ms.

**Fig. 8:**
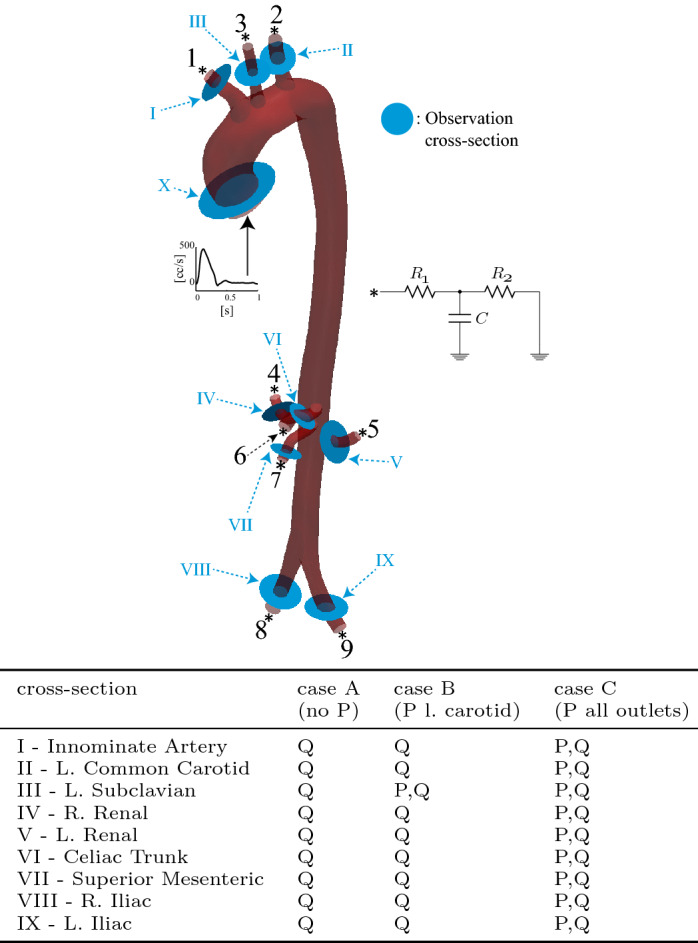
Boundary conditions, outlet numbering, and observation planes (depicted in blue, roman numerals) in the subject-specific aorta with synthetic data. The table shows the different sets of observations considered. In all cases, flow was observed at every branch. In case A, no pressure observations were available. Case B used a single pressure observation in the left carotid (outlet III). In Case C, pressure was observed in every branch

*Forward problem* Synthetic data was generated using known parameter values for the Windkessel LPN in all nine outlets. The simulation was run to full periodicity, then cross-sectional averaged pressure and volumetric flow waveforms in the aortic branches were recorded during the final cardiac cycle, at 160 Hz; see Fig. 8 for the locations at which these observations were made.

*Estimation problem* Motivated by the difficulty of obtaining pressure data in the clinic, we examined three different estimation cases, each with differing availability of pressure data. Case A considered no pressure data, Case B used a single pressure observation at the left carotid artery, and Case C assumed pressure observations in every outlet vessel. In all three cases, flow data in every outlet vessel were available; this choice is justified due to the much greater availability of flow data in the clinic. A key assumption in all three cases was that the initial state variables, including pressure, were assumed to be error-free; the impact of this assumption will be addressed later in this section.

Sequential estimations were then run for each case. Initial estimation error covariances were all set to $s_i=0.2$, and measurement error covariances for pressure and flow to $w_P^{(i)}=(29.3\hbox { mmHg})^2$ and $w_Q^{(i)}=(8.3\hbox { cc/s})^2$, respectively (see Appendix: Measurement error covariance matrix). The synthetic data were interpolated linearly to provide values for all time-steps of the simulation.

**Fig. 9:**
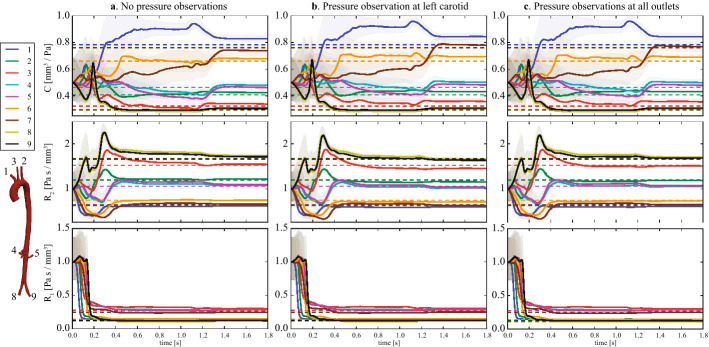
Subject-specific aorta with synthetic data: evolution of Windkessel parameters during estimation, assuming error-free initial state variables. (Left) Case A: estimation with no pressure observations. (Middle) Case B: estimation run with a single pressure observation in the left carotid artery (cross-section III in Fig. 8). (Right) Case C: estimation run with a pressure observation in each of the branches (see Fig. 8). From top to bottom: Windkessel compliance, *C*, proximal resistance, $R_1$, and distal resistance, $R_2$. Dashed lines denote the true parameter value used in the forward simulation and the solid lines the estimate. The shaded regions cover the standard deviation of the parameter estimates

Results for cases A, B and C are presented in Fig. 9, demonstrating the evolution of the twenty-seven Windkessel parameters over 2.0 s (one cardiac cycle $\sim 0.8$ s). Stable parameter estimates were achieved in all cases. However, while $R_1$ converged rapidly (third row plots), *C* and $R_{2}$ took longer to converge (first and second row plots, respectively).

The final estimated values are summarized in Table 1, where the errors are stratified into three categories; those with errors in excess of 5% (colorless cells), those with less than 5% error (blue cells), and those with less than 2.5% error (green cells). For the resistance estimates $R_1$ and $R_2$, there is little difference between Cases B and C, and both are superior to Case A. This indicates that having at least one pressure observation is beneficial. Conversely, for the compliance estimates *C*, there is little difference between Cases A and B, and both are inferior to Case C. This suggests that reconstruction of Windkessel compliances in multi-branched models benefits from having pressure measurements in more than one location, something it is not always available in the clinic. Regardless, in this example we note that the relative errors are generally low in all cases, suggesting that, *given reasonable initial guesses for the parameters and the model state*, parameter identifiability does not strongly depend on pressure data.

**Table 1:** Final estimated Windkessel parameters

Outlet	1	2	3	4	5	6	7	8	9
*C* ($\hbox {mm}^3$/Pa)									
True values	0.783	0.409	0.324	0.464	0.464	0.660	0.761	0.293	0.296
A (no P)	0.844	0.432	0.358	0.500	0.480	0.693	0.775	0.302	0.308
% error	7.83%	5.64%	10.31%	7.78%	**3.38%**	**4.96%**	*1.84%*	**2.98%**	**4.16%**
B (1 P)	0.843	0.431	0.354	0.498	0.475	0.690	0.765	0.302	0.308
% error	7.73%	5.38%	9.26%	7.23%	*2.44%*	**4.54%**	*0.50%*	**2.91%**	**4.13%**
C (all P)	0.829	0.425	0.340	0.480	0.463	0.679	0.739	0.302	0.308
% error	5.88%	**3.76%**	**4.73%**	**3.40%**	− *0.12%*	**2.97%**	− **2.87%**	**2.85%**	**4.02%**
$R_2$ (Pa s/$\hbox {mm}^3$)									
True values	0.629	1.202	1.517	1.060	1.061	0.746	0.647	1.679	1.664
A (no P)	0.598	1.157	1.444	1.018	1.034	0.714	0.629	1.652	1.627
% error	− textbf4.93%	− **3.76%**	− **4.83%**	− **3.94%**	− **2.55%**	− **4.29%**	− **2.78%**	− *1.57%*	− *2.22%*
B (1 P)	0.615	1.194	1.507	1.055	1.073	0.736	0.650	1.706	1.680
% error	− *2.17%*	− *0.65%*	− *0.70%*	− *0.47%*	*1.18%*	− *1.34%*	*0.47%*	*1.61%*	*0.97%*
C (all P)	0.625	1.219	1.539	1.079	1.096	0.748	0.659	1.733	1.708
% error	− *0.59%*	*1.38%*	*1.46%*	*1.77%*	**3.31%**	*0.29%*	*1.83%*	**3.27%**	**2.67%**
$R_1$ (Pa s/$\hbox {mm}^3$)									
True values	0.137	0.137	0.269	0.279	0.279	0.147	0.246	0.107	0.122
A (no P)	0.137	0.144	0.306	0.290	0.288	0.147	0.253	0.114	0.128
% error	*0.32%*	5.21%	14.05%	**3.89%**	**3.28%**	*0.05%*	**2.62%**	6.25%	**4.71%**
B (1 P)	0.137	0.144	0.306	0.290	0.288	0.148	0.252	0.114	0.127
% error	*0.04%*	**4.67%**	14.00%	**3.83%**	**3.28%**	*0.40%*	*2.43%*	5.90%	**4.42%**
C (all P)	0.135	0.141	0.302	0.287	0.286	0.148	0.248	0.111	0.125
% error	− *1.27%*	**2.77%**	12.31%	**2.59%**	**2.56%**	*0.68%*	*0.83%*	**3.69%**	**2.56%**

Case A has no pressure data, Case B has pressure data only in the left carotid artery, and Case C has pressure data at all nine outlets of the model (c.f. Fig. 8). See Fig. 9 for the parameter evolution in each case. Errors less than 5% in absolute value are highlighted in bold; those less than 2.5% are shown in italic

**Fig. 10:**
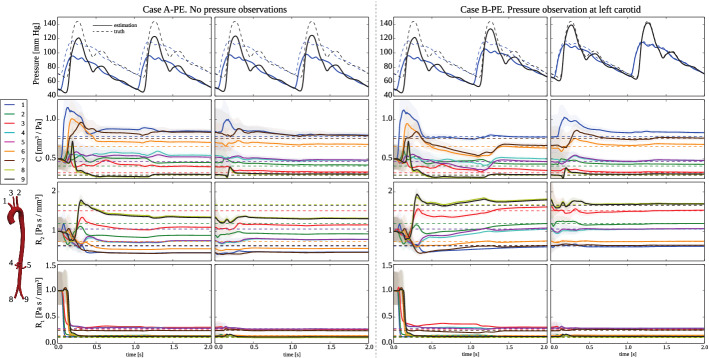
Subject-specific aorta with synthetic data: evolution of Windkessel parameters during estimation with initial errors introduced into the pressure field. (Left) Case A-PE: estimation run with no pressure observations. (Right) Case B-PE: estimation run with a single pressure observation in the left carotid artery (cross-section III in Fig. 8). From top to bottom: pressure waveforms at outlets 1 (innominate artery, blue lines) and 9 (left common iliac, black lines); Windkessel compliance, *C*; proximal resistance, $R_1$; and distal resistance, $R_2$. The dashed lines denote the truth and the solid lines the estimate. The shaded regions cover the standard deviation of the parameter estimates. In both cases, estimations were run for two cardiac cycles, stopped, and then started again using as initial estimates the results of the first run

*Estimation with initial errors in the pressure field* To examine the importance of accurate initial states, Cases A and B were performed a second time, now with an initial uniform error of 20 mmHg in the pressure field. We refer to these as the “pressure error” (PE) Cases, A-PE and B-PE. From top to bottom, Fig. 10 shows estimation and true data for pressure at outlets 1 (innominate artery, blue lines) and 9 (left common iliac artery, black lines), and estimates of compliance *C*, distal resistance $R_{2}$ and proximal resistance $R_{1}$. In both cases, estimation were run for two cardiac cycles, stopped, and then started again using as initial estimates the results of the first run. We observed that in Case A-PE the parameter values can not be recovered, particularly, the distal resistance values, $R_2$. However, the results for Case B-PE show that a single pressure observation is sufficient to enable parameter recovery, even with errors in the initial pressure field. Note that in this case, Fig. 10 shows that the pressure waveforms in case B-PE agree closely with the data and that the parameters are recovered. The percentage parameter errors in each case are given in Appendix: Parameter errors With and without pressure data.

**Fig. 11:**
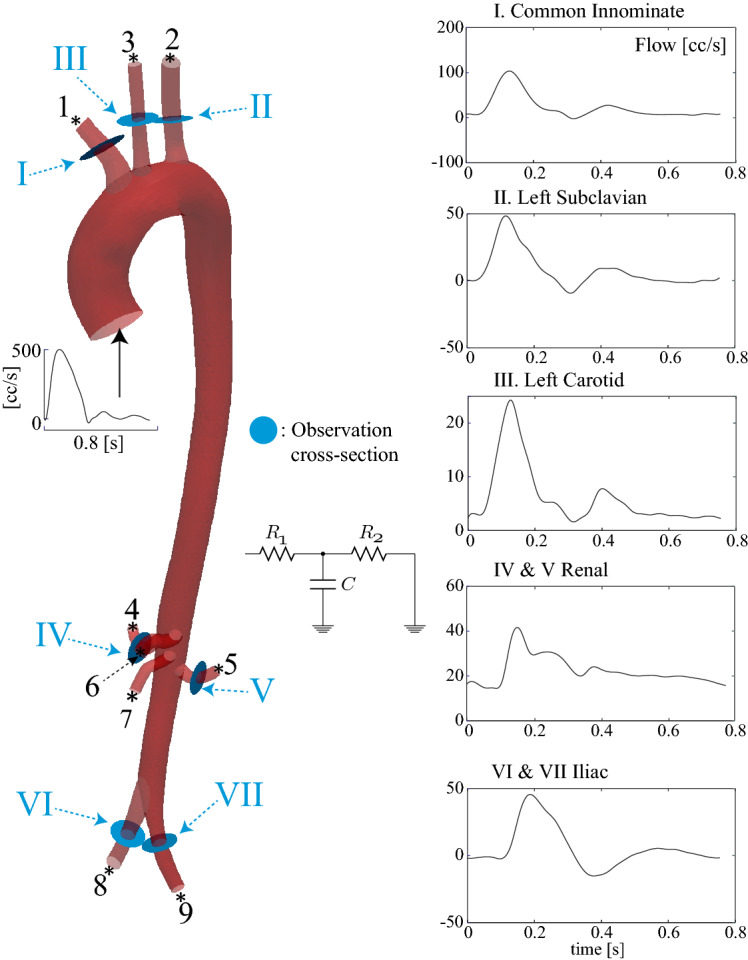
Left: the flow observation sites (roman numerals) in the simulated aorta, which coincide with the flow acquisition planes in the PCMRI scans. Waveforms at oulets IV and VI were not measured directly with MRI, but were assumed to be identical to those measured at sites V and VII, respectively. Parameters at outlets 4 (celiac trunk) and 5 (superior mesenteric artery) were not estimated due to the lack of flow data. For simulation, three-element Windkessel models are attached at each outlet, indicated by the asterisks. Right: flow waveforms derived from PCMRI data were used in the estimation problem

**Fig. 12:**
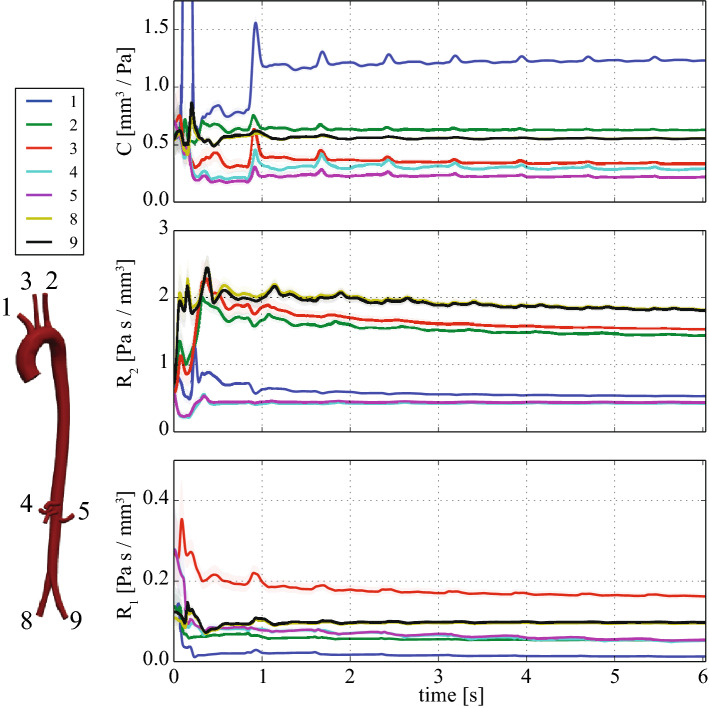
Subject-specific aorta with PC-MRI and applanation tonometry data: evolution of Windkessel parameters during estimation for each outlet. Top: compliance *C*; middle: distal resistance $R_2$; bottom: proximal resistance $R_1$. The solid lines denote the estimated values for each parameter; the shaded regions show the range of estimated values plus/minus the the estimation error standard deviation, providing a visual representation of the estimation error

**Fig. 13:**
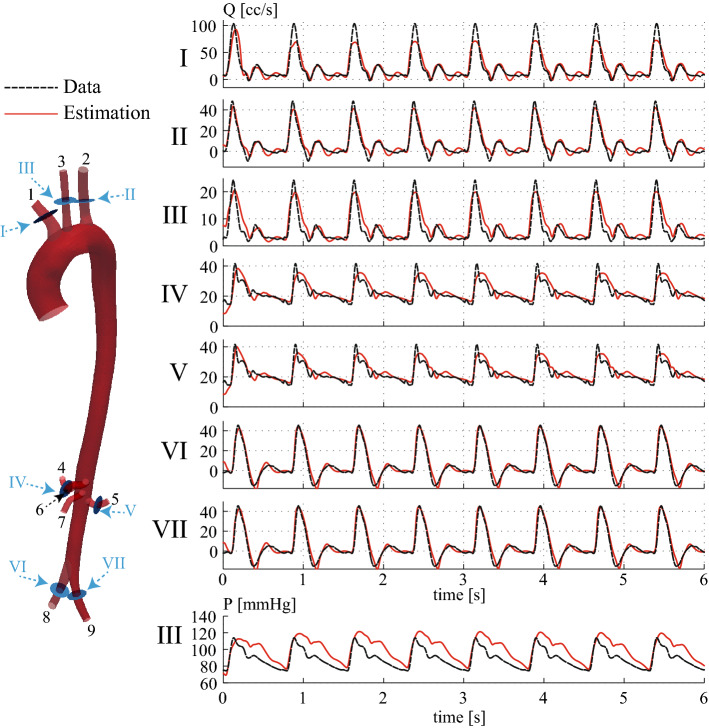
Subject-specific aorta with PC-MRI and applanation tonometry data: observations from the model (red lines), computed using the *a posteriori* estimate during the estimation procedure, compared with the measured data (black lines) at each of the seven observation locations for flow, and the location for pressure (outlet III)

**Fig. 14:**
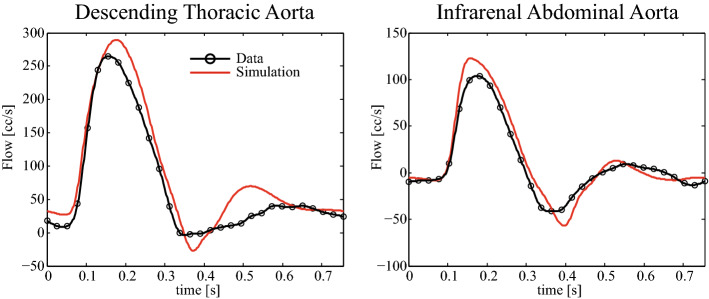
Volumetric flow in the descending aorta (left), and the infrarenal abdominal aorta (right) during a forward simulation using the final estimated parameters (see Fig. 12). The waveforms are compared to the PC-MRI data

### Subject-specific aorta with PC-MRI and applanation tonometry data

We studied the same aortic geometry, now using subject-specific PC-MRI flow and applanation tonometry pressure data. The flow data were recorded in the ascending aorta, the three branches of the aortic arch, the left renal and the left iliac arteries, and two other locations within the aorta. Right renal and right iliac flow data were synthesized as copies of those for the left renal and left iliac. Observation locations in the model were chosen to agree with the PC-MRI acquisition planes, as shown in Fig. 11. Pressure data consisted of the cycle-averaged pressure waveform acquired in the left carotid artery. Details on the data acquisition are given in “Data acquisition” section.

*Estimation problem* Windkessel parameters were estimated at all but two outlets; the celiac trunk (outlet 6) and the superior mesenteric artery (outlet 7) were omitted due to the absence of local flow data. Thus, estimates of $R_1^{i}$, $R_2^{i}$, and $C^{i}$, $i\in \{1, \dots , 7\}$, were made; a total of twenty-one parameters.

The initial guess for $R_1^{(i)}$, for each *i*, was chosen to match the characteristic impedance of the associated 3D outlet [44]. Initial values for $R_{2}^{(i)}$ were chosen such that $R_1^{(i)}+R_2^{(i)}$ was the same at all estimated outlets, and such that the mean pressure at the known inflow rate was physiological. The initial guesses for $C^{(i)}$ were based on an estimation of the total peripheral compliance [45, 46], and apportioned to each outlet following previous work [7]. These initial parameter values are given in Table 4. The Windkessel parameters at the celiac trunk and mesenteric artery, which were not subject to estimation, are given in Table 5. The measurement error covariances were chosen to be $w_P=(75\hbox { mmHg})^2$ for the pressure observations, and $w_Q^{(i)}=(20\hbox { cc/s})^2$ for the flow observations.

The estimation problem was run. Figure 12 shows the evolution and convergence of the Windkessel parameters over eight cardiac cycles. The parameters are seen to be generally stable after the first cycle is completed, with minimal subsequent adjustments.

The true parameters in this subject-specific case are unknown, so evaluation of the results must be made in terms of the agreement with flow and pressure data. Such an evaluation is made in Fig. 13, where we observe that the simulated flow rates initially differ significantly from the data, before rapidly converging, so that the flow waveforms match the overall shape of the data at the various flow observation sites. The best agreement is observed in the aortic arch branches (outlets I, II, and III) and iliac branches (outlets VI and VII), whereas the biggest discrepancies in the flow waveforms are observed in the renal arteries (outlets IV and V). Figure 13 also compares the predicted left carotid pressure waveform (outlet III) with the subject data. Results revealed that the simulation matches well with the tonometry data during the early part of systole, but shows some discrepancy during the diastolic phase.

Figure 14 shows two comparisons between predicted and subject-recorded flow waveforms in two locations; one in the descending thoracic aorta, and the other in the infrarenal abdominal aorta. These data were not provided to the filter during parameter estimation, so the observed agreement provides stronger validation of the method. Each predicted waveform was generated using the final estimated parameters, as shown in Table 2.

**Table 2:** Final estimates after eight cardiac cycles for the Windkessel parameters for the subject-specific aortic aorta estimation problem with PC-MRI and applanation tonometry data

Outlet	$R_1$ (Pa s/$\hbox {mm}^3$)	*C* ($\hbox {mm}^3$/Pa)	$R_2$ (Pa s/$\hbox {mm}^3$)
1 (innominate)	0.0127	1.23	0.532
2 (subclav., left)	0.0524	0.628	1.44
3 (carotid, left)	0.162	0.334	1.53
4 (renal, right)	0.0515	0.293	0.421
5 (renal, left)	0.0547	0.220	0.441
8 (iliac, right)	0.0948	0.555	1.82
9 (iliac, left)	0.0981	0.553	1.81

### Reduced order Unscented Kalman Filter with constrained least squares (ROUKF-CLS)

In this section, we consider two estimation problems made to test the numerical performance of the ROUKF-CLS algorithm. These problems utilize simple geometries and flow and pressure assumptions which are not always of high physiological relevance but that nevertheless provide a solid testbed for our purposes.

#### ROUKF-CLS verification against ROUKF using a Windkessel model

We compared the ROUKF and ROUKF-CLS methods by estimating the parameters of a three-element Windkessel. We applied the ROUKF-CLS using a Netlist-implemented Windkessel (i.e. specified using the arbitrary boundary condition framework of Equation ). Conversely, we used the ROUKF to estimate the parameters in an equivalent “hard-coded” Windkessel, implemented in terms of Eq. 3. This test enabled us to determine equivalence of the estimation algorithms in cases where either method is applicable, and also verify the correctness of the Netlist filtering implementation.

*Forward problems* In both cases, we considered a deformable vessel, 40 mm in length and 5.9 mm in diameter, with a pulsatile velocity boundary condition of period $\hbox {T} = 1.1$ s at the inlet. The vessel stiffness and thickness were $E = 0.7$ MPa and $h = 0.3$ mm, respectively. In the ROUKF method, a hard-coded Windkessel model was coupled at the outlet, whereas in the ROUKF-CLS method, a Netlist Windkessel model was used. The vessel was discretized into 4157 elements, and the time-step was 1 ms. The simulation was run until full periodicity was achieved, and spatially-averaged pressure and volumetric flow waveforms were recorded on an observation plane at the center of of the vessel.

**Fig. 15:**
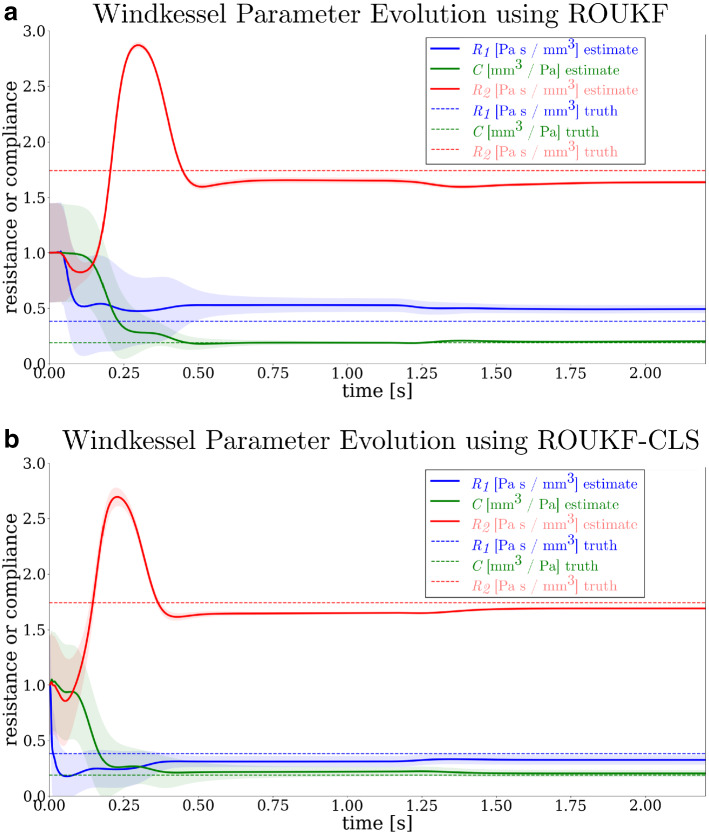
Comparison between Windkessel parameter estimation using different filtering methods. **a** Case A, with ROUKF, using the non-augmented filtering algorithm and the “hard-coded” Windkessel model **b** Case B, with ROUKF-CLS, using the constrained least squares augmentation of ROUKF, and the Netlist Windkessel model. Convergence of the parameter values for the three LPN parameters is shown. Given uniform initialization of all parameters to 1, the true values (dashed lines) are recovered. Shaded regions indicate the standard deviations for the parameter estimates, demonstrating the evolving confidence of the filter in the estimate

*Estimation problems* In each method, we set the initial value of all parameters to be estimated to 1.0, and attempted to recover the observed pressure and flow data. The measurement error covariances were chosen to be $w_P=(10\hbox { mmHg})^{2}$ for the pressure observations, and $w_Q^{(i)}=(1\hbox { cc/s})^{2}$ for the flow observations. Initial estimation error covariances were all set to $s_i=0.6$. In both cases, the parameter evolution followed similar trajectories, reaching good estimates within one second of simulation time, as shown in Fig. 15. This result strongly supports the equivalence of the two algorithms for estimating Windkessel LPN.

#### ROUKF-CLS applied to a coronary LPN model

**Fig. 16:**
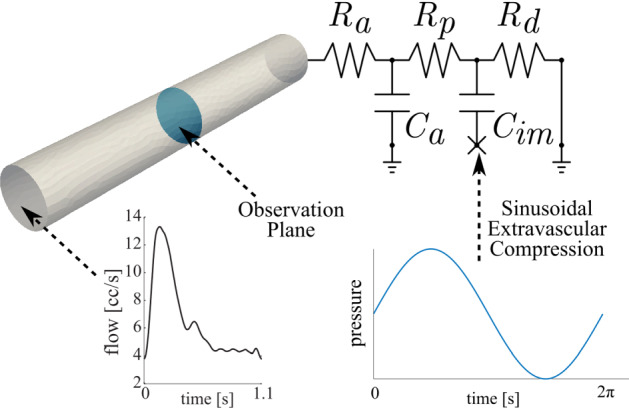
Illustration of the problem setup for filtering the five-element coronary LPN using ROUKF-CLS. The inflow velocities are prescribed, and the outflow face is coupled to a Netlist coronary model. The artificial sinusoidal extravascular compression waveform is shown, applied to the base of capacitor $C_{im}$

We next studied the estimation of all five parameters in a single coronary LPN model, whose structure is shown in Fig. 16 [25]. As described in “Estimation algorithm—ROUKF-CLS for arbitrary LPNs” section, the ROUKF-CLS method is required due to the multiple internal states of this LPN. Here, we considered a rigid vessel, 68.9 mm in length and 9.6 mm in diameter. At the inflow, a generic pulsatile velocity condition of period $\hbox {T}=1.1$ s was imposed, and the coronary LPN was attached at the outflow. A sinusoidal time-varying pressure of period $\hbox {T}=2\pi$ s was applied to the base of the coronary capacitor $C_{im}$. This represents a synthetic extravascular compression, and is imposed to demonstrate numerical efficacy of ROUKF-CLS in the presence of such a load on the LPN.

*Forward problem* The simulation was run using known parameter values and a time-step of 10 ms for a total of 34 cardiac cycles, capturing sufficient variations in solution due to the different periods of the inflow and the intra-myocardial pressure wavefroms. Synthetic data, consisting of spatially-averaged pressure and volumetric flow, was collected at a cross-sectional plane located in the center of the vessel.

**Fig. 17:**
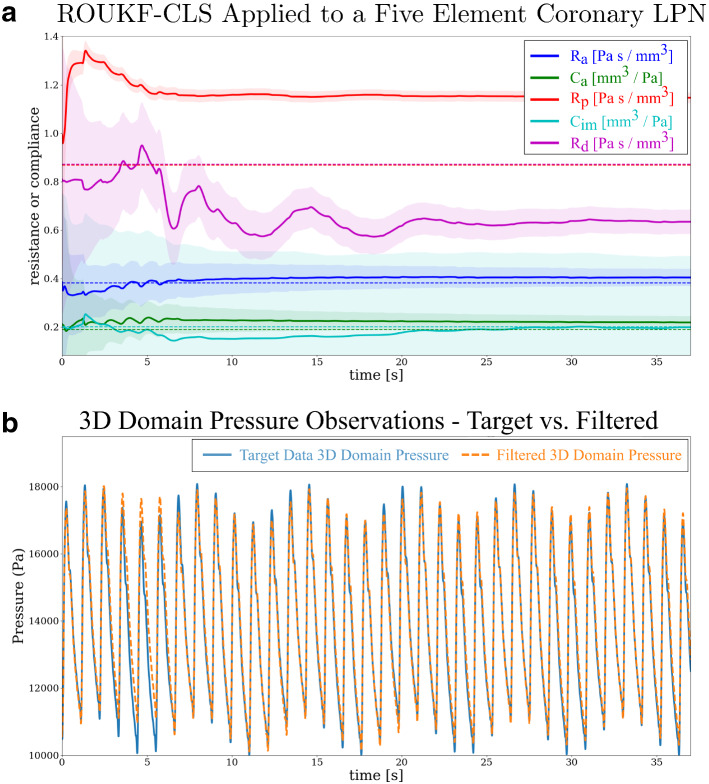
**a** Filtering of the five coronary LPN parameters. The true value of each parameter during synthetic data generation is shown by dashed lines; colors indicate which component each belongs to. The units of resistances are $\mathrm {Pa}~\mathrm {s}~\mathrm {mm}^{-3}$, and the units of compliance are $\mathrm {mm}^{3}~\mathrm {Pa}^{-1}$. Shaded regions show the standard deviations of the filtered estimates. **b** Target pressure observations at the cross-sectional plane and the sequentially achieved pressure observations during the filtering process

*Estimation problem* All five coronary LPN parameters were initialized to 1, and The measurement error covariances were chosen to be $w_P=(10\hbox { mmHg})^{2}$ for the pressure observations, and $w_Q^{(i)}=(1\hbox { cc/s})^{2}$ for the flow observations. Initial estimation error covariances were all set to $s_i=0.3$. The sequential estimation simulation was run for 34 cardiac cycles. Similarly to the results in case F of “Estimation of Windkessel parameters from pressure and flow” section, where estimation of all three Windkessel parameters was made, parameter estimates for the coronary LPN did not converge fully after the first estimation run (results not shown here). Therefore, the filtering procedure was restarted from the final parameter states of the first estimation run. Figure 17 presents the results of this second sequential estimation run; Fig. 17a shows the convergence of the coronary LPN parameters to steady values, and Fig. 17b demonstrates that the observed pressure waveform converges to that of the synthetic target data, as desired. Note the expected sinusoidal variation in the peak and minimum pressures, due to the externally-imposed waveform. Fig. 17a shows that some of the original parameter values are not recovered. In particular, $R_p$ and $R_d$ overestimated and underestimated, respectively, their true values (denoted by the superimposed dashed magenta and red lines in the graph at $y=0.86$) by an equal amount. Note that this implies that the recovered value of $R_p + R_d$ is correct, but that the individual parameters were not identifiable. This will be discussed further in “ROUKF-CLS filtering of a LPN coronary Netlist model” section.

## Discussion

We discuss the most relevant findings of the different results presented earlier, in particular for the subject-specific aortic case (with both synthetic data and also PC-MRI and applanation tonometry data), and also for the tests examining the numerical performance of the ROUKF-CLS algorithm.

### Subject-specific aorta cases

#### Subject-specific aorta with synthetic data

This application example included two main estimation scenarios: (1) parameter estimation assuming error-free initial states (flow and pressure), and (2) parameter estimation assuming errors for the initial state variables.

The first scenario is unrealistic for any clinical application. Nevertheless, under the major assumption of error-free initial states, we could see that reasonable results were obtained for the estimated parameters even if no pressure observation was available (Case A in Table 1). Of course, having a perfect knowledge of the pressure initial state (since it was assumed error-free) is indeed an important observation in itself. Interestingly, Case B (a single pressure observation available, a rather realistic clinical scenario) revealed that the largest error in the estimated compliance corresponded to the vessel with the pressure observation. Errors for the compliance estimates consistently smaller than 5% could only be achieved for Case C, which assumed pressure observations in all branches, a highly unusual clinical scenario.

As expected, when errors in the initial state were introduced (see Fig. 10), we observed that the Windkessel parameters could not be recovered if no pressure observation was available (Case A-PE).

#### Subject-specific aorta with PC-MRI and applanation tonometry data

A key result of this paper was demonstrating that Windkessel parameters can be recovered for a subject-specific model of the human aorta and its main branches, using PC-MRI flow data for each outlet, together with a single applanation tonometry pressure recording (“Subject-specific aorta with PC-MRI and applanation tonometry data” section). This is of particular interest for clinical applications, as non-invasive flow measurements can be readily acquired, whereas the options for non-invasive pressure measurement are limited and of lower fidelity.

The model observation flow waveforms shown in Fig. 13 displayed good agreement with the measured data, once the Windkessel parameters have stabilized, demonstrating that the parameter estimates are good. However, for the pressure data, a clear discrepancy in the diastolic decay phase is noticeable. This may be due to the fact that flow and pressure were not acquired simultaneously in the subject. Furthermore, the vessel of interest (left common carotid in this case) is compressed slightly during the applanation tonometry procedure, introducing a wave reflection site that is simply not present in the PC-MRI flow waveforms. One could therefore argue that not only are the flow and pressure waveforms not acquired simultaneously, but that they correspond to different hemdoynamic conditions altogether. Therefore, the estimator will give more weight to the flow or the pressure data, depending on the choices made for the measurement error covariances. However, it is clear from Case B-PE in Fig. 10 that when flow and pressure data are acquired simultaneously—at least in a synthetic case— the pressure waveforms can also be closely recovered.

For additional validation, we compared the simulation results with subject-specific flow data which was not used during the estimation procedure. In Fig. 14, we observed that, using the final estimated parameters, the simulated flow and the unseen data agreed well. Note that there is a good match of the more predominant diastolic backflow in the infrarenal abdominal aorta, compared to the milder diastolic backflow in the descending thoracic aorta. These results provide additional confidence in the value and efficacy of the methods.

An interesting observation is that the estimated $R_1$ parameter in the upper branches of the aorta differed significantly from the initial guesses, which were determined by theoretically matching the characteristic impedances at the outlets. This implies that this common method for assigning $R_1$, which fundamentally minimizes wave reflections at the outlet, may not always be appropriate.

### ROUKF-CLS algorithm

#### ROUKF-CLS validation against ROUKF by application to a three element Windkessel model

In order to test the ROUKF-CLS algorithm, we evaluated it on a problem to which both ROUKF and ROUKF-CLS can be applied: that of estimating three-element Windkessel parameters. The two cases, one with ROUKF (Case A) and ROUKF-CLS (Case B), differed in two aspects. The first is whether the implementation of the Windkessel LPN was hard coded (Case A) or via Netlist (Case B); this aspect is unrelated to filtering. The second aspect is which of ROUKF or ROUKF-CLS was used as the filtering algorithm. The reason for this dual difference is that ROUKF-CLS is designed for filtering arbitrary LPNs; creation of these requires Netlist functionality. The results shown in Fig. 15 thus provide validation of both the Netlist implementation, and the ROUKF-CLS algorithm. In this particular case, it is arguable that the ROUKF-CLS (Fig. 15b) produces better results. This gives us confidence in the efficacy of the formulation.

The ROUKF algorithm is applicable to Windkessel models because they are sufficiently simple; ROUKF-CLS is a generalization to a wider class of LPNs.

#### ROUKF-CLS filtering of a LPN coronary Netlist model

This case allowed us to validate ROUKF-CLS in a more complex setting. The parameters of the five-element LPN coronary model were shown to converge to stable values in Fig. 17a. Strikingly, despite the differences from the ground-truth parameter values, the stable values give a good reproduction of the data (Fig. 17b). This perhaps indicates that, with only one pressure and one volumetric flow recording, the LPN parameters are not uniquely identifiable. This identifiability issue may also be reflected by the fact that the standard deviations—the shaded regions which indicate the algorithm’s confidence in the parameter estimates—remain relatively large in some cases. Future work should confirm this in a formal parameter identifiability study [16].

The errors in the final estimates for the five LPN components $R_{a}$, $C_{a}$, $R_{p}$, $C_{im}$ and $R_{d}$ were respectively 5.8% 15.7% 31.8% 1.1% 27.1%. At face value, this indicates some substantial errors, but this would seem at odds with the high-accuracy recovery of the pressure recording shown in Fig. 17b. Looking more carefully, we see that the filter has has recovered the correct *total* resistance; the sum of the estimates $R_{a}+R_{p}+R_{d}=2.184$ and the true total resistance is 2.122, giving an overall error of 2.9% in the resistance. For the two more distal resistors, the sum of the estimates is 1.781, and the true sum is 1.740; this is 2.4% a error. Similarly, for the total compliance, the sum of the estimates $C_{a}+C_{im}=0.416$, and the true sum is 0.389, giving an error of 6.9%. This supports the hypothesis that the parameters are not uniquely identifiable with the available data.

### The impact of constrained least squares

**Fig. 18:**
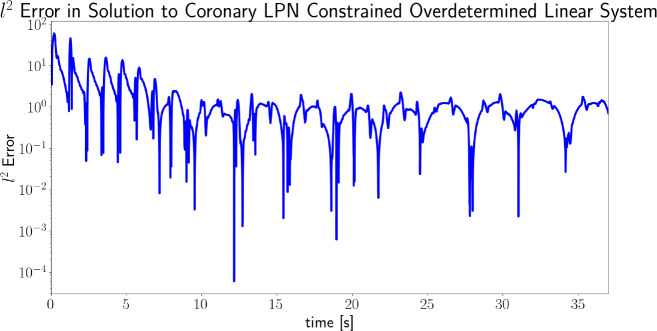
Log plot of the $\ell ^{2}$ norm of the error between the constrained least squares solution and the desired right-hand side for the coronary LPN case, as given by $\left\| L_{LPN}^{over} {\mathbf {x}} - {\mathbf {b}}\right\|$ (cf. Eq. 18). The data is presented over time, demonstrating how the magnitude of the perturbations required to enforce LPN consistency reduces over time, as the parameters converge. The actual parameter values over time for this simulation are shown in Fig. 17

Figure 18 shows the $\ell ^{2}$ error (Eq. 18) between the solution to the CLS system and the overdetermined target for the LPN coronary model results of Fig. 17. This measures the extent to which CLS is adjusting the system to maintain LPN consistency as the parameters are adjusted. As the parameters converge, the CLS system comes close to being solved exactly and therefore little adjustment is taking place on the system.

**Fig. 19:**
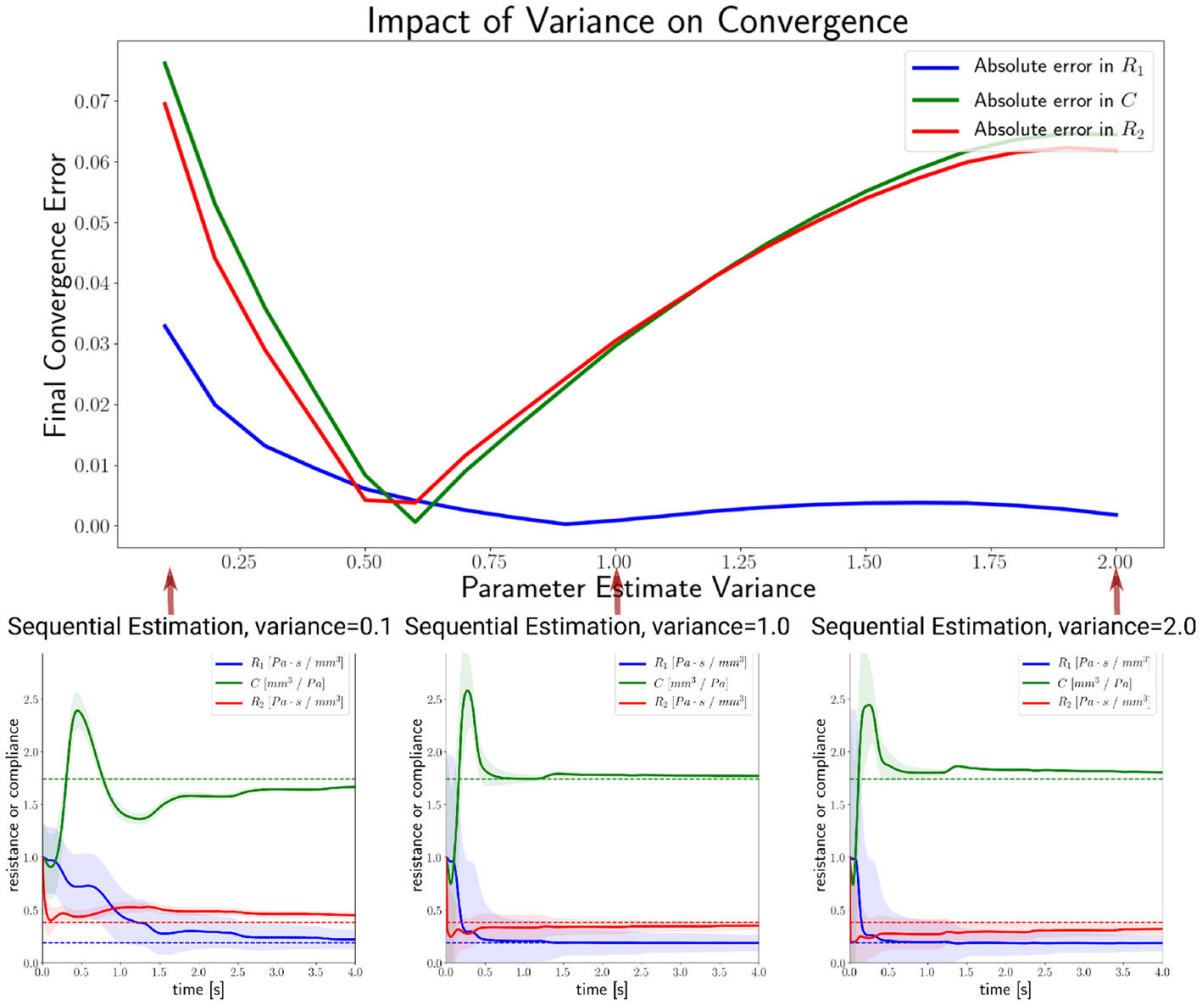
Upper panel: the absolute error in each of the three Windkessel parameters after 4 seconds of estimation simulation, at different values of initial parameter estimate covariances $s_{i}$ (set uniformly for all three parameters). Lower three images: the parameter convergence history with the initial parameter estimate variances set to 0.1, 1.0 and 2.0 (left to right). See “The choice of initial covariance values for the estimated parameters” section for further discussion

### An empirical strategy for resolving convergence failures

In general, we would expect that good initial guesses for the values of most parameters are possible, based on an understanding of the parameters in models of similar patients. When initial guesses are poor, there is a chance that convergence will fail. However, as seen in “Estimation of Windkessel parameters from pressure and flow” and “ROUKF-CLS applied to a coronary LPN model” sections, we found that poor convergence could be resolved by restarting the estimation simulation, using the final estimated parameters from the previous simulation. We speculate that this resolution is due to the following. Whilst the filter can adjust the parameters, it is constrained by the time history of the simulation, and—regardless of further adjustments to the parameters—it may have reached a state (i.e. pressure, velocity and wall displacement in the 3D domain) too far from the expected pressure and flow limit cycle to ever converge back to it. Restarting the simulation resets the state, and so permits convergence of the parameters. This could be done systematically, for example by choosing to always reset the state, keep the parameters, and restart the estimation at the end of every cardiac cycle.

### The choice of initial covariance values for the estiamted parameters

We generally used initial covariance values of $s_{i}=0.2$ for the parameter estimates—with some variation between cases—regardless of the parameter or its order of magnitude. The values chosen gave good convergence, whether assessed by the recovery of a known synthetic value, or in terms of recovery of the data itself with the converged parameters. To explore this in more detail, Fig. 19 shows the impact of different initial parameter covariance choices on the parameter estimates recovered after 4 s of estimation, for the same problem as was presented in “ROUKF-CLS verification against ROUKF using a Windkessel model” section using the ROUKF-CLS method. To evaluate longer-term behaviour, these estimation simulations were run for longer, and the results demonstrate that for some variance choices, convergence is slower. The minimal variation in the ultimate results (see Fig. 19, lower plots) indicate that the initial variance choice is not of critical importance, and that the values used in the present work represent reasonable choices.

### The impact of the choice of measurement error covariance

**Fig. 20:**
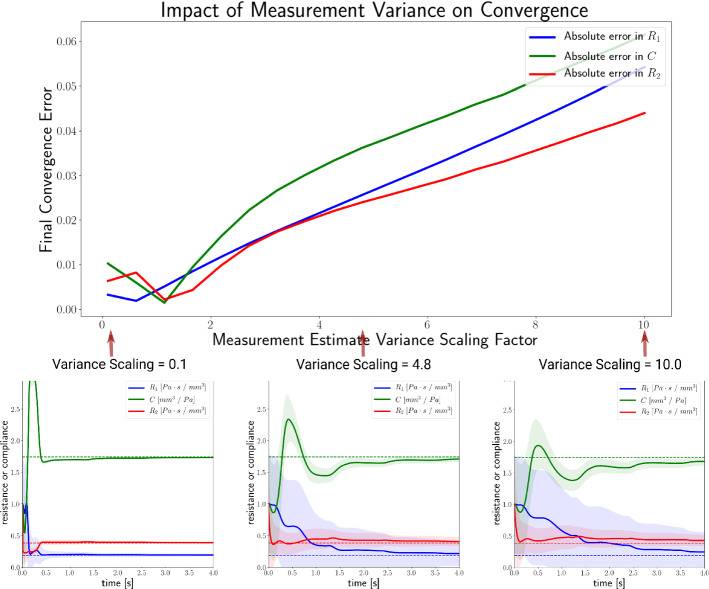
Upper panel: the absolute error in each of the three Windkessel parameters after 4 s of estimation simulation, at different scalings of measurement (pressure and flow) covariances (scaled multiplicatively and uniformly for both). Baseline values are as given in “ROUKF-CLS verification against ROUKF using a Windkessel model” section Lower three images: the parameter convergence history with the measurement variances, $w_P^{(i)}$ and $w_Q^{(i)}$, scaled by 0.1, 4.8 and 10.0 (left to right). See “The impact of the choice of measurement error covariance” section for further discussion

The measurement error covariance for the pressure and flow data, $w_P^{(i)}$ and $w_Q^{(i)}$ respectively, are chosen according to how much we trust the measurements. In the present work, we chose these such that the square root of the variance was around 10% of the total variation in the measured quantity. To explore the impact of this choice on the convergence of the parameter estimates, we multiplicatively scaled the covariances by various values between 0.1 and 10.0, running an estimation simulation for each. The ROUKF-CLS Windkessel case of “ROUKF-CLS verification against ROUKF using a Windkessel model” section. is used as a baseline (scaling factor = 1.0). The errors in the parameter estimates after 4 s of estimation are plotted against the scaling factor in the upper panel of Fig. 20. Three of the resulting parameter convergence plots are shown in the lower portion of the figure. We observe that increasing the trust in the data (reducing the variance that we assert for the data) increases the speed of convergence. This is unsurprising in this case, as the measurement data is synthetic. We expect that optimisation of the measurement covariance for clinical data would be dependent on the particular data source, and likely needs to be determined once for each type of data source and device model.

### Computational cost

The total computational cost of the ROUKF procedure scales linearly with the number, $N$, of parameters to be estimated; specifically, the cost is $N+1$ times the cost of a forward simulation. It is easily parallelized, as once the $N+1$ particles are generated for each time-step, they represent $N+1$ independent simulations.

An additional strategy for reducing the computational cost is to use a low-fidelity model (a coarse simulation mesh, or a reduced model such as 1D Navier–Stokes) to obtain reasonable parameter estimates rapidly, which can then be fine-tuned via short simulations using the high-resolution model.

## Conclusion

This work studied the application of Kalman filtering techniques for automatic determination of parameters in computational hemodynamics models. The three main contributions of this paper are:

1. A demonstration of automatic determination of boundary condition parameters in a subject-specific, 3D FSI model of the human aorta and its main branches. An efficient sequential filtering technique, the ROUKF, was used to assimilate blood flow and pressure waveform data. We found that successful estimation of all three Windkessel parameters at every outlet is possible when the data consists of one flow observation at each outlet together with at least a single pressure observation. A key aspect is that the data used can be readily recordable in the clinic. The most powerful demonstration of this is given in Fig. 13.
2. We demonstrated that the ROUKF can also be used to simultaneously estimate regional wall material properties and Windkessel parameters, by using a distance operator to incorporate wall motion data, in conjunction with a pressure observation. The key results were given in Fig. 6.
3. For cases where the boundary condition models are more complex than three-element Windkessel LPNs, we introduced a modified filtering procedure, the ROUKF-CLS, which augments ROUKF to enable estimation of parameters in arbitrary LPN boundary condition circuits. We demonstrated this in the case of a five-element coronary LPN boundary condition. The key result here was shown in Fig. 15. ROUKF-CLS greatly expands the class of computational hemodynamics problems to which the ROUKF can be applied. An implementation of ROUKF-CLS was developed and tested as part of the Netlist arbitrary boundary condition design system in the computational hemodynamics software package CRIMSON.

## Figures and Tables

**Fig. 21:**
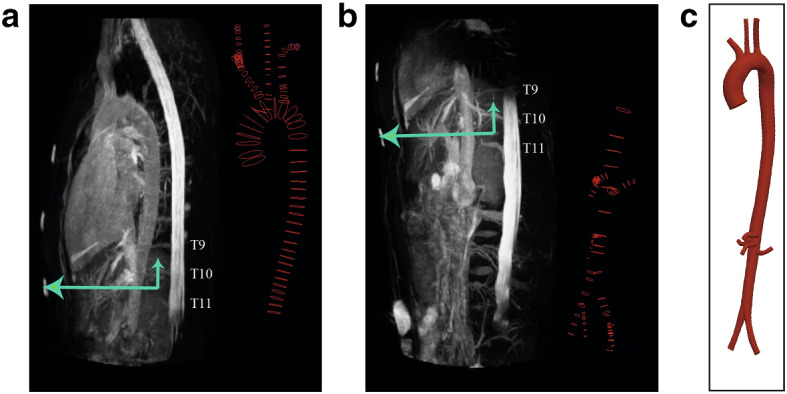
**a** The upper thoracic image, containing the aortic arch and neck vessels. **b** The lower thoracic image, containing the aortic bifurcation. Two-dimensional segmentations (red) perpendicular to vessel centerlines. Normal vectors (cyan), common to both images, were defined using the chest ECG electrode and two intervertebral discs as fiducial markers. **c** The 3D geometry was created by interpolating a parametric surface from the 2D segmentations

## Data Availability

The datasets used and/or analysed during the current study are available from the corresponding author on reasonable request.

## Appendix

### MRI data acquisition and geometric segmentation

A free-breathing SSFP sequence with fat-suppression was used to acquire anatomy images of the aorta covering its full extent from the arch down to the aortic bifurcation. Two separate image volumes were obtained in parasagittal orientation, one above and one below the diaphragm. ECG-triggering and respiratory navigation were employed to avoid motion artifacts and to ensure a good spatial resolution of $1.2 \times 1.2 \times 1.8$ mm. To extract the subject-specific geometry of the aorta, the above-diaphragm and below-diaphragm images were segmented separately to generate 2D contours perpendicular to the centerlines of the aorta and its first generation of branches. To spatially align the two sets of contours, a rigid body transformation was defined using visible elements common to both images as fiducial markers: the center of the inferior ECG electrodes (connection fluid) and the centers of two intervertebral discs (located between the thoracic vertebrae T11 and T10, and between T10 and T9) were used to generate an orthonormal set of vectors for each image volume (Fig. 21) and thus a transformation for the below-diaphragm contours. The combined set of 2D contours was used to generated a 3D parametric surface, which in turn was used to create the finite element meshes.

PC-MRI data was obtained with a temporal resolution of 35 ms and spatial resolution of $1.04 \times 1.04$ mm. The acquisition planes were placed orthogonal to the direction of flow. Velocity encoding was adapted to the maximum velocity in the different vessels (80–200 cm/s) to ensure accurate measurement of velocity profiles. Static tissue phase correction was employed to correct for potential baseline errors using the vendor’s software. The area in each individual vessel segment was computed and the phase images were processed to obtain time-resolved flow waveforms using the commercial software package GTFlow (GyroTools LLC). A cubic spline interpolation was then performed to resample the flow data with a 2 ms resolution.

### Pressure data acquisition

Pressure data was obtained at the carotid arteries using applanation tonometry. The sampling rate was 128 Hz and the pressure measurements were calibrated using systolic and diastolic pressures obtained from a pressure cuff (Omron Healthcare). The pressure and PC-MRI flow data were not acquired simultaneously; however, the phase lag between pressure and flow was derived by time-shifting the flow and pressure waveforms relative to each other so that a linear relationship between pressure and flow during early systole is achieved [47].

### Sigma point generation

The $N+1$ spherical simplex sigma point directions $\sigma$ are generated recursively as follows, using the procedure given by [36]. We initialize the $\sigma$ vectors as follows:

24 $$\begin{aligned} \sigma _{(1)}^{1}&= \frac{-1}{2\alpha _{\mathrm{s}}^{(1)}} \ \ \ \sigma _{(2)}^{1} = \frac{1}{2\alpha _{\mathrm{s}}^{(2)}} \end{aligned}$$

where the sigma point weights, $\alpha _{\mathrm{s}}^{(i)}$, are given by:

25 $$\begin{aligned} \alpha _{\mathrm{s}}^{(i)} = \frac{1}{N+1}, i = 1,\ldots ,N+1 \end{aligned}$$

Then, for $j=2,\ldots ,N$ and $1 \le i \le j+1,$

26 $$\begin{aligned} \sigma _{(i)}^{j} = \left\{ \begin{array}{ll} \left[ \begin{array}{c}\sigma _{(i)}^{j-1} \\ \\ \frac{-1}{\sqrt{j(j+1)\alpha _{\mathrm{s}}^{(i)}}}\end{array}\right] &{} i = 1,\ldots ,j \\ \\ \left[ \begin{array}{c}0_{j-1} \\ \frac{j}{\sqrt{j(j+1)\alpha _{\mathrm{s}}^{(i)}}}\end{array}\right]&i = j + 1 \end{array} \right. \end{aligned}$$

Here, $0_{j}$ is a column vector containing *j* zeros.

### Measurement error covariance matrix

In the examples presented, the measurement error covariance matrix at time step *k*, $W_k$, was assumed to be a constant diagonal matrix of the form:

27 $$\begin{aligned} W_k = \left[ \begin{array}{cccccc} w_Q^{(1)} &{} 0 &{} &{} \ldots &{} &{} 0 \\ 0 &{} \ddots &{} &{} &{} &{} \\ &{} &{} w_Q^{n_\text {obs-Q}} \\ \vdots &{} &{} &{} w_P^{(1)} &{} &{} \vdots \\ &{} &{} &{} &{} \ddots &{} \\ 0 &{} &{} \ldots &{} &{} &{} w_P^{n_\text {obs-P}} \end{array} \right] , \end{aligned}$$

where $w_Q^{(i)}$ corresponds to the *i*-th flow observation location and $w_P^{(j)}$ corresponds to the *j*-th pressure observation location. Diagonal entries for other observations, such as wall motion, are included as necessary.

### Parameter errors with and without pressure data

Table 3 compares the percentage errors in each estimated parameter in the case where an initial pressure error is present in the model, and in the presence (Case A-PE) or absence (Case B-PE) of pressure data recorded at a single location on the vasculature. It can be seen that the results are greatly superior in the presence of the pressure measurement. This study was described in “Subject-specific aorta with synthetic data” section.

**Table 3:** Estimated Windkessel parameters (at the end of two cardiac cycles) in the cases with an initial error in the pressure field (corresponding to Fig. 10).

Outlet	1	2	3	4	5	6	7	8	9
*C* ($\hbox {mm}^3$/Pa)									
True values	0.783	0.409	0.324	0.464	0.464	0.660	0.761	0.293	0.296
A-PE (no P)	0.800	0.422	0.333	0.481	0.485	0.683	0.793	0.304	0.309
% error	*2.20%*	**3.19%**	**2.72%**	**3.56%**	**4.63%**	**3.56%**	**4.25%**	**3.85%**	**4.40%**
B-PE (1 P)	0.833	0.431	0.331	0.479	0.480	0.683	0.763	0.305	0.309
% error	6.39%	5.25%	*1.92%*	**3.15%**	**3.52%**	**3.56%**	*0.27%*	**4.01%**	**4.57%**
$R_2$ (Pa s/$\hbox {mm}^3$)									
True values	0.629	1.202	1.517	1.060	1.061	0.746	0.647	1.679	1.664
A-PE (no P)	0.478	0.938	1.165	0.796	0.799	0.568	0.468	1.336	1.322
% error	− 24.0%	− 22.0%	− 23.2%	− 24.9%	− 24.7%	− 23.8%	− 27.6%	− 20.4%	− 20.6%
B-PE (1 P)	0.621	1.200	1.535	1.064	1.069	0.749	0.649	1.707	1.693
% error	− *1.27%*	− *0.15%*	*1.18%*	*0.41%*	*0.77%*	*0.39%*	*0.41%*	*1.67%*	*1.75%*
$R_1$ (Pa s/$\hbox {mm}^3$)									
True values	0.137	0.137	0.269	0.279	0.279	0.147	0.246	0.107	0.122
A-PE (no P)	0.137	0.136	0.263	0.273	0.273	0.143	0.240	0.108	0.122
% error	*0.32%*	− *0.75%*	− *2.21%*	− *2.35%*	− *2.16%*	− **2.69%**	− **2.66%**	*0.26%*	− *0.20%*
B-PE (1 P)	0.143	0.142	0.278	0.285	0.286	0.153	0.255	0.116	0.130
% error	**4.90%**	**3.59%**	**3.53%**	*2.18%*	**2.73%**	**4.14%**	**3.39%**	7.67%	6.58%

Cases A-PE and B-PE are identical to Cases A and B of Table 1 respectively, except for the introduction of an initial error in the pressure field. Errors less than 5% in absolute value are highlighted in bold; those less than 2.5% are shown in italic

### Initial parameter guesses

**Table 4:** Initial guesses for the Windkessel parameters for the full aorta estimation problem with real data

Outlet	$R_1$ (Pa s/$\hbox {mm}^3$)	*C* ($\hbox {mm}^3$/Pa)	$R_2$ (Pa s/$\hbox {mm}^3$)
1	0.1366	0.5618	0.7166
2	0.1373	0.5624	0.7159
3	0.2686	0.6887	0.5846
4	0.2794	0.7017	0.5738
5	0.2786	0.7007	0.5746
8	0.1073	0.5398	0.7459
9	0.1220	0.5506	0.7312

$R_1$ is matched to the characteristic impedance of the outlet vessel

**Table 5:** Windkessel parameters that are fixed and not estimated in the full aorta estimation problem with real data

Outlet	$R_1$ (Pa s/$\hbox {mm}^3$)	*C* ($\hbox {mm}^3$/Pa)	$R_2$ (Pa s/$\hbox {mm}^3$)
6	0.14709	0.4862	0.82796
7	0.24618	0.5524	0.72887

Tables 4 and 5 list the initial parameter values used in the aorta estimation with real data, presented in “Subject-specific aorta with PC-MRI and applanation tonometry data ” section.

## Abbreviations

$$\vec {v}$$: Fluid velocity
$$\vec {u}$$: Solid displacement
$\vec {\dot{v}}$: Time derivative of fluid velocity
$$p$$: Fluid pressure
$$\rho _\text {f}$$: Fluid density
$$\rho _\text {s}$$: Solid density
$\mu$: Fluid dynamic viscosity
$\vec {w}$: Test function for momentum conservation equation
$q$: Test function for continuity equation
$$\Omega ^\text {f}$$: Fluid domain
$$\Gamma _{\text {in}}$$: Inflow boundary
$$\Gamma _{\text {out}}$$: Outflow boundary
$\Gamma _{\text {w}}$: Wall boundary/interface
$\vec {n}_\text {f}$: Outward normal vector on fluid domain boundary
$\varvec{\tau }_\text {f}$: Fluid viscous stress
$\varvec{\sigma }_\text {s}$: Solid domain Cauchy stress
$\varvec{\tilde{K}}$: Tensor of material parameters
$\varvec{P}$: Wall pre-stress tensor
$\varvec{\epsilon }$: Wall strain tensor
$\mathbf {E}$: Linearized wall stiffness
$\mathbf {k}$: Wall transverse shear factor
$$\nu $$: Poisson’s ratio
$\mathcal {S}$: Solution space for velocity
$\mathcal {P}$: Solution space for pressure
$\mathcal {W}$: Momentum test function space
$$\hat{X}$$: State estimate
$$\hat{\theta }$$: Parameter estimate
$P$: Estimation error covariance
$W$: Measurement error covariance
$-$: Denotes *a priori* estimate
$+$: Denotes *posteriori* estimate
$Z$: The measurement term in Kalman filtering
$L$: ROUKF covariance factor *L*
$U$: ROUKF covariance factor *U*
$$H$$: Observation operator
$A$: Model forward operator
$\Gamma$: The innovation term in Kalman filtering
$\{HL\}$: Observed state sensitivity
$N$: Size of the reduced state (i.e. the parameters)
$\sigma$: Sigma point vector
$[\sigma _{(*)}]$: Matrix whose columns are the sigma point vectors
$\alpha _\text {s}$: Sigma point weight
$D_\alpha$: Diagonal matrix of sigma point weights
$v$: Fluid velocity nodal solution vector
$\dot{v}$: Fluid acceleration nodal solution vector
$p$: Pressure numerical nodal solution vector
$u$: Displacement numerical nodal solution vector

## Footnotes

1 For a normal (non-filtering) simulation, this system could be immediately used as part of an iterative scheme to determine the coefficients ${\tilde{R}}$ and ${\tilde{S}}$ for Equation . This is the core of the Netlist boundary condition system.

2 This is the reason for why ROUKF-CLS is not required for a three-element Windkessel; it has exactly one such row, so requires no CLS augmentation.
